# Supplementary material for: Identification of 13 Novel Loci in a Genome-Wide Association Study on Taiwanese with Hepatocellular Carcinoma
Source: Int J Mol Sci. 2023 Nov 16;24(22):16417. doi: 10.3390/ijms242216417 (PMC10671380; doi:10.3390/ijms242216417)
Supplement: Supplementary file 1 [file ijms-24-16417-s001.zip › ijms-2669820-supplementary.pdf]

Identification of 13 Novel Loci in A Genome-Wide Association Study on Taiwanese with Hepatocellular Carcinoma

Table of contents

Fig S1. ..……………………………………………………………………………………………...1

Fig S2. ..……………………………………………………………………………………………...2

Fig S3. 3……………………………………………………………………………………………...3

Fig S4. ..……………………………………………………………………………………………16

Fig S5. ..……………………………………………………………………………………………17

Fig S6. ..……………………………………………………………………………………………18

Fig S5. ..……………………………………………………………………………………………19

Table S1……………………………………………………………………………………………20

Table S2……………………………………………………………………………………………20

Table S3……………………………………………………………………………………………20

Table S4……………………………………………………………………………………………21

Table S6

Table S6.1…………………………………………………………………………………22

Table S6.2…………………………………………………………………………………22

Table S7

Table S7.1…………………………………………………………………………………23

Table S7.2…………………………………………………………………………………23

Table S7.3…………………………………………………………………………………24

Table S7.4…………………………………………………………………………………..24

Table S7.5…………………………………………………………………………………..25

Table S7.6………………………………………………………………………………….....26

Table S7.7………………………………………………………………………………….....27

Table S7.8………………………………………………………………………………….....28

Table S7.9………………………………………………………………………………….....28

Table S7.10…………………………………………………………………………………29

Table S7.11……29
Table S7.12……30
Table S7.13……30
Table S7.14……30
Table S7.15……31
Table S7.16……31
Table S7.17……32
Table S7.18……32
Table S7.19……33
Table S7.20……34
Table S7.21……34
Table S7.22……35
Table S7.23……35
Table S7.24……36
Table S8
Table S8.1……37
Table S8.2……37
Table S8.3……38
Table S9……38
Table S10……38

Fig S1. Regional plots and R-square of linkage disequilibrium for 13 novel loci. The blue solid line shows the recombination rate, the gray horizontal dash line indicates a significant threshold ($p = 5e-08$), and the LD score with the target variant is colored on each variant. (A) rs187199523. (B) rs140233124. (C) rs144285059. (D) rs148610742. (E) rs118180127. (F) rs117719091. (G) rs17155112. (H) rs77404202. (I) rs144225287. (J) rs150098717. (K) rs74333160. (L) rs80115676. (M) rs61404501.

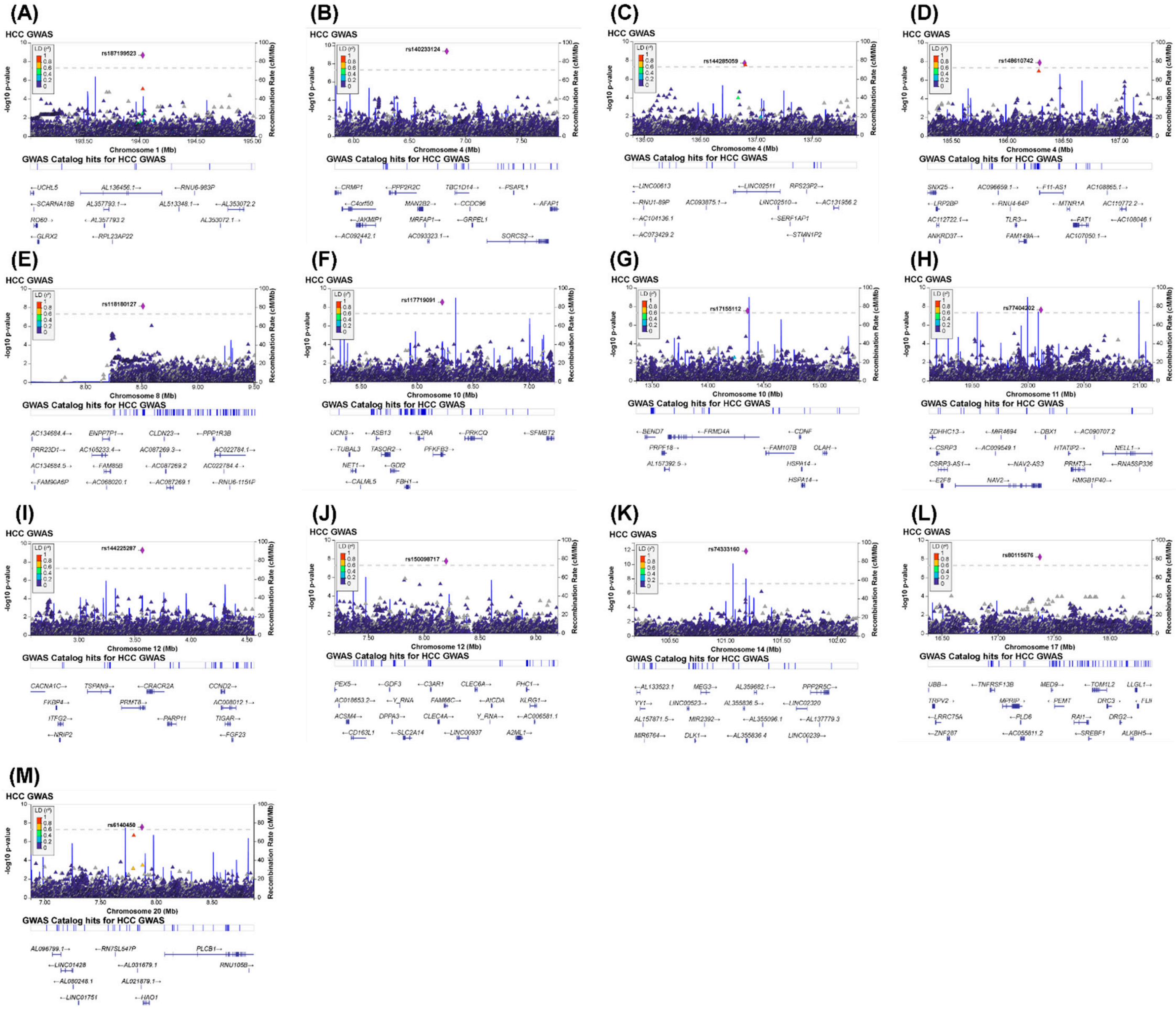

Figure S2. Results of the GWAS for Taiwanese patients with HCC in another cohort. Manhattan plot adjusted by sex and age and QQ-plot in another cohort in Taiwanese.

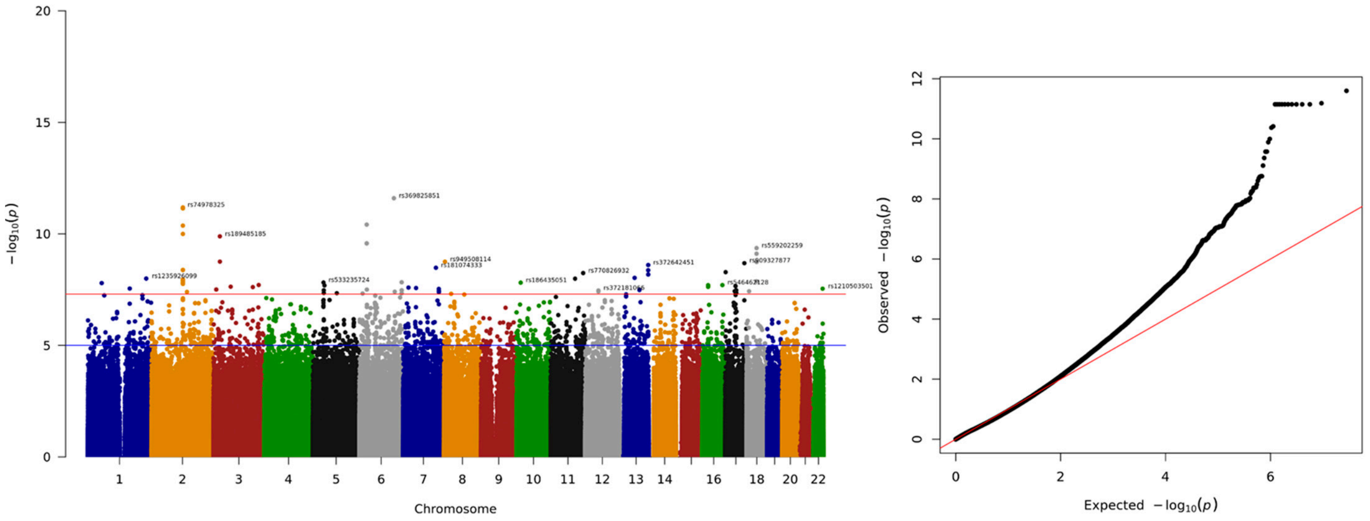

Figure S3. PheWAS Manhattan plot for 13 novel loci in Taiwanese with HCC. The negative log of the p-value is plotted for each of 1,791 disease phenotypes or "phecodes" in CMUH. Each color represents a disease category as defined on the x-axis. (A) rs187199523. (B) rs140233124. (C) rs144285059. (D) rs148610742. (E) rs118180127. (F) rs117719091. (G) rs17155112. (H) rs77404202. (I) rs144225287. (J) rs150098717. (K) rs74333160. (L) rs80115676. (M) rs61404501.

(A)

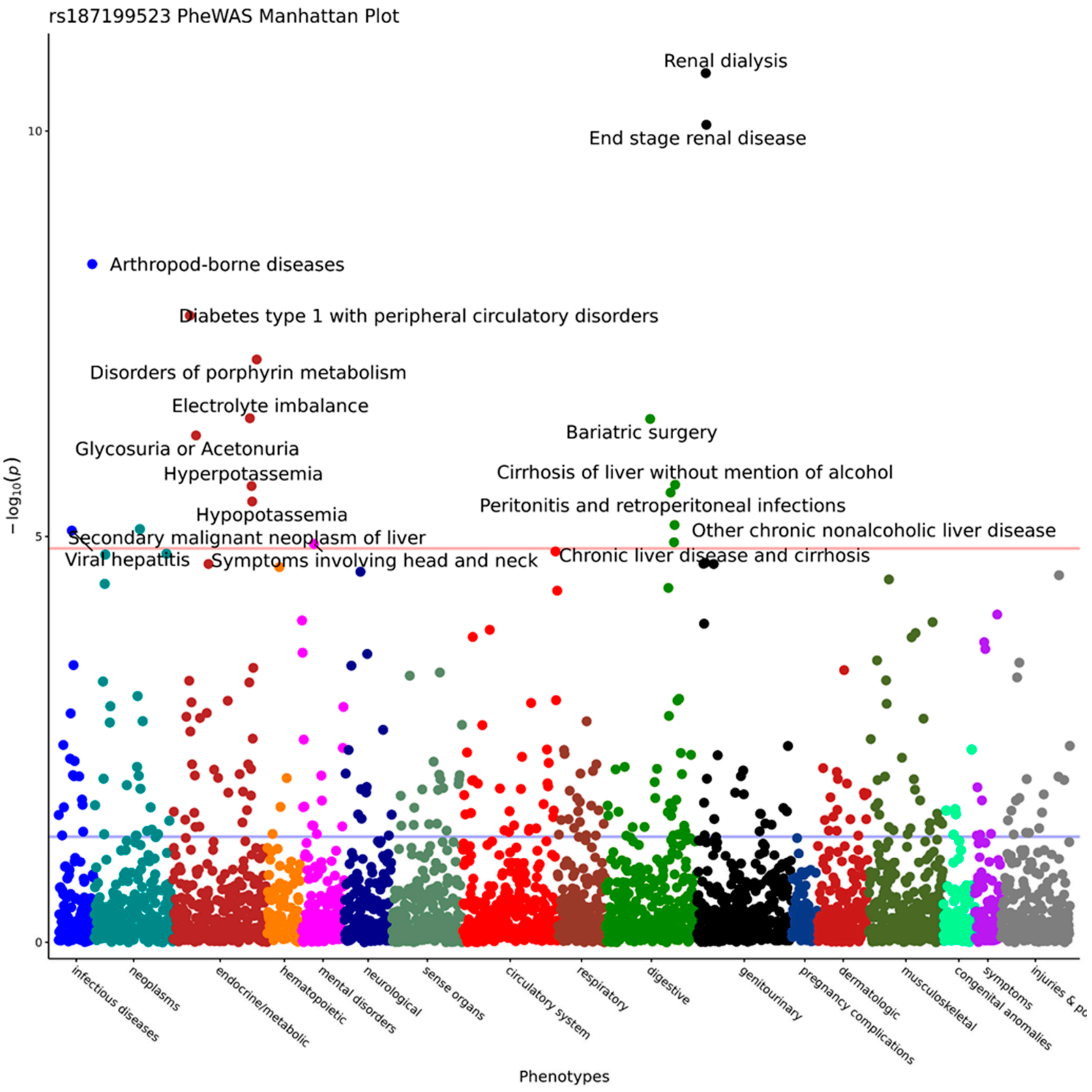

(B)

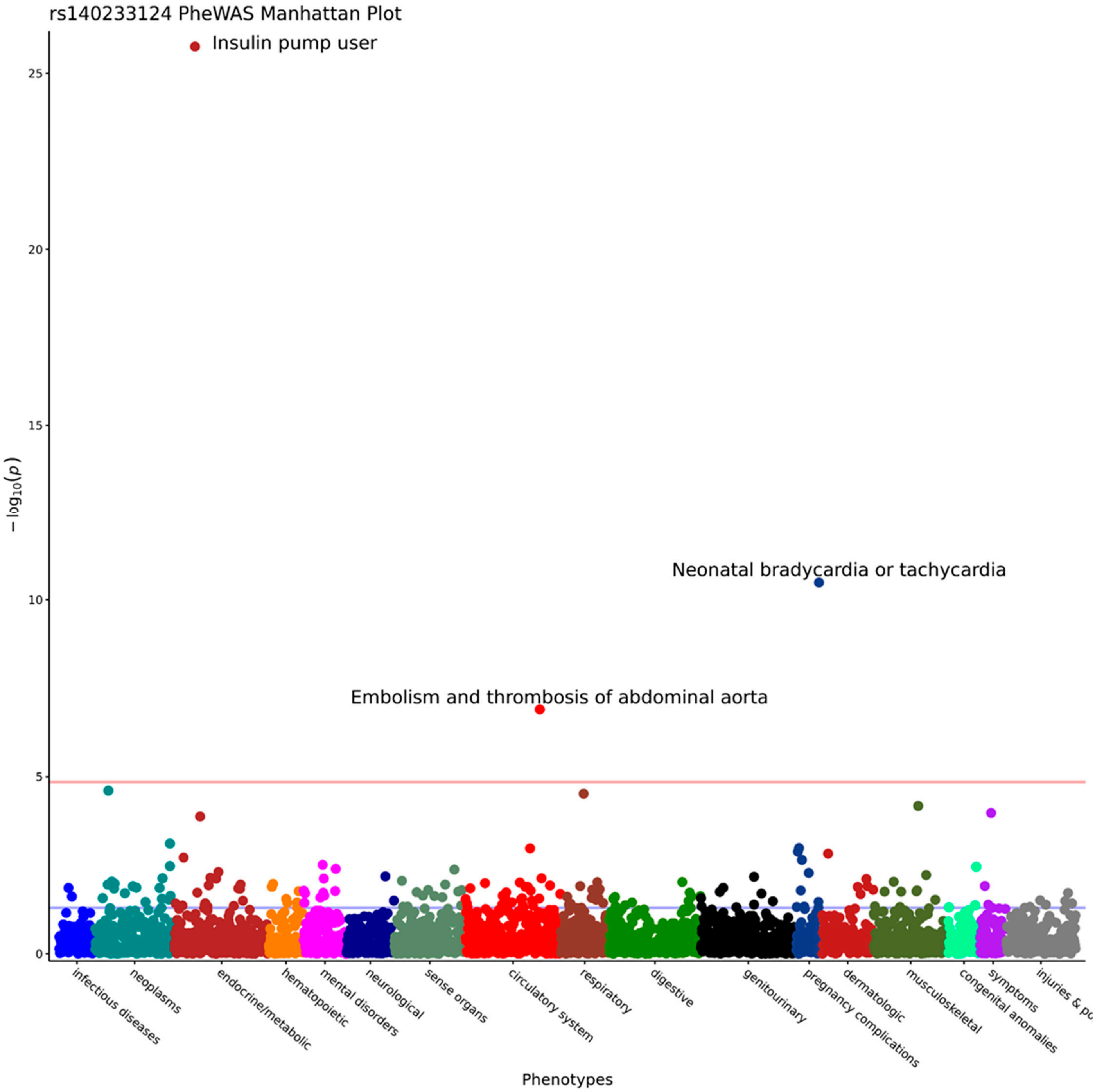
rs140233124 PheWAS Manhattan Plot
Insulin pump user
Neonatal bradycardia or tachycardia
Embolism and thrombosis of abdominal aorta
$-\log_{10}(p)$
0
5
10
15
20
25
infectious diseases
neoplasms
endocrine/metabolic
hematopoietic
mental disorders
neurological
sense organs
circulatory system
respiratory
digestive
genitourinary
pregnancy complications
dermatologic
musculoskeletal
congenital anomalies
symptoms
Phenotypes

(C)

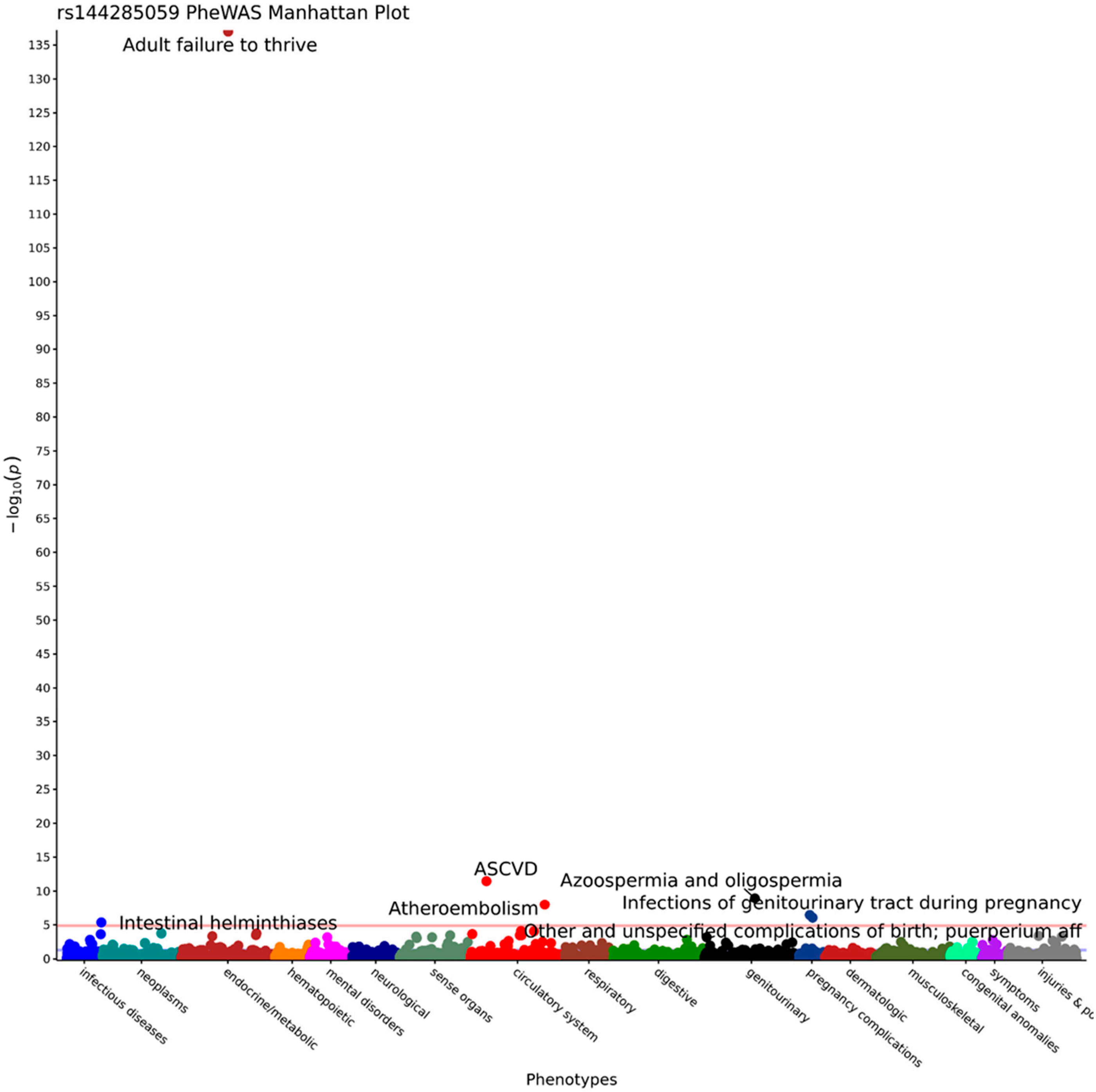

rs144285059 PheWAS Manhattan Plot
Adult failure to thrive
ASCVD
Azoospermia and oligospermia
Atheroembolism
Infections of genitourinary tract during pregnancy
Intestinal helminthiases
Other and unspecified complications of birth; puerperium aff
$-\log_{10}(p)$
0
5
10
15
20
25
30
35
40
45
50
55
60
65
70
75
80
85
90
95
100
105
110
115
120
125
130
135
infectious diseases
neoplasms
endocrine/metabolic
hematopoietic
mental disorders
neurological
sense organs
circulatory system
respiratory
digestive
genitourinary
pregnancy complications
dermatologic
musculoskeletal
congenital anomalies
symptoms
Phenotypes

(D)

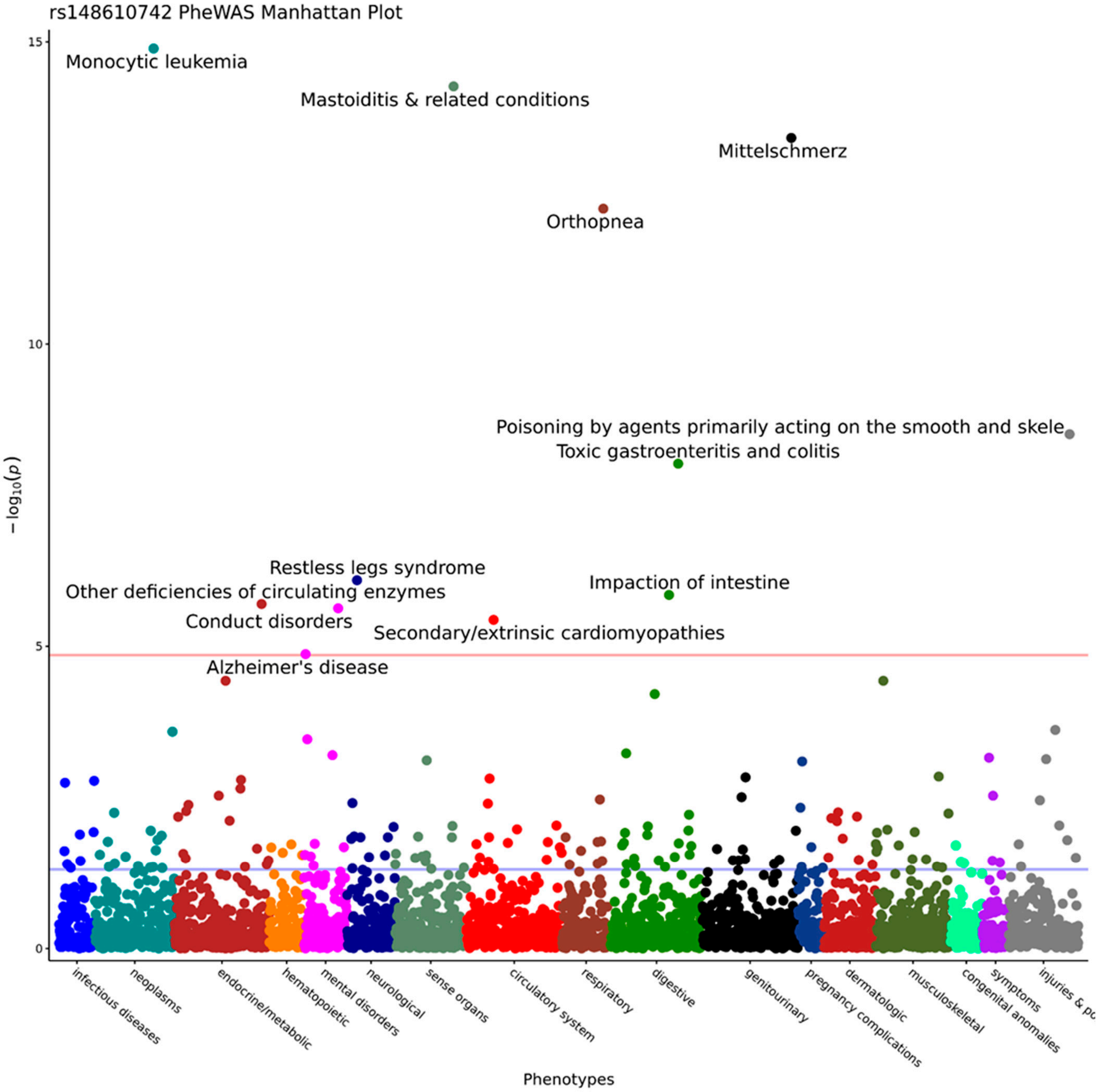
rs148610742 PheWAS Manhattan Plot
Monocytic leukemia
Mastoiditis & related conditions
Mittelschmerz
Orthopnea
Poisoning by agents primarily acting on the smooth and skele
Toxic gastroenteritis and colitis
Restless legs syndrome
Other deficiencies of circulating enzymes
Impaction of intestine
Conduct disorders
Secondary/extrinsic cardiomyopathies
Alzheimer's disease
15
10
5
0
− log10(p)
infectious diseases
neoplasms
endocrine/metabolic
hematopoietic
mental disorders
neurological
sense organs
circulatory system
respiratory
digestive
genitourinary
pregnancy complications
dermatologic
musculoskeletal
congenital anomalies
symptoms
Phenotypes

(E)

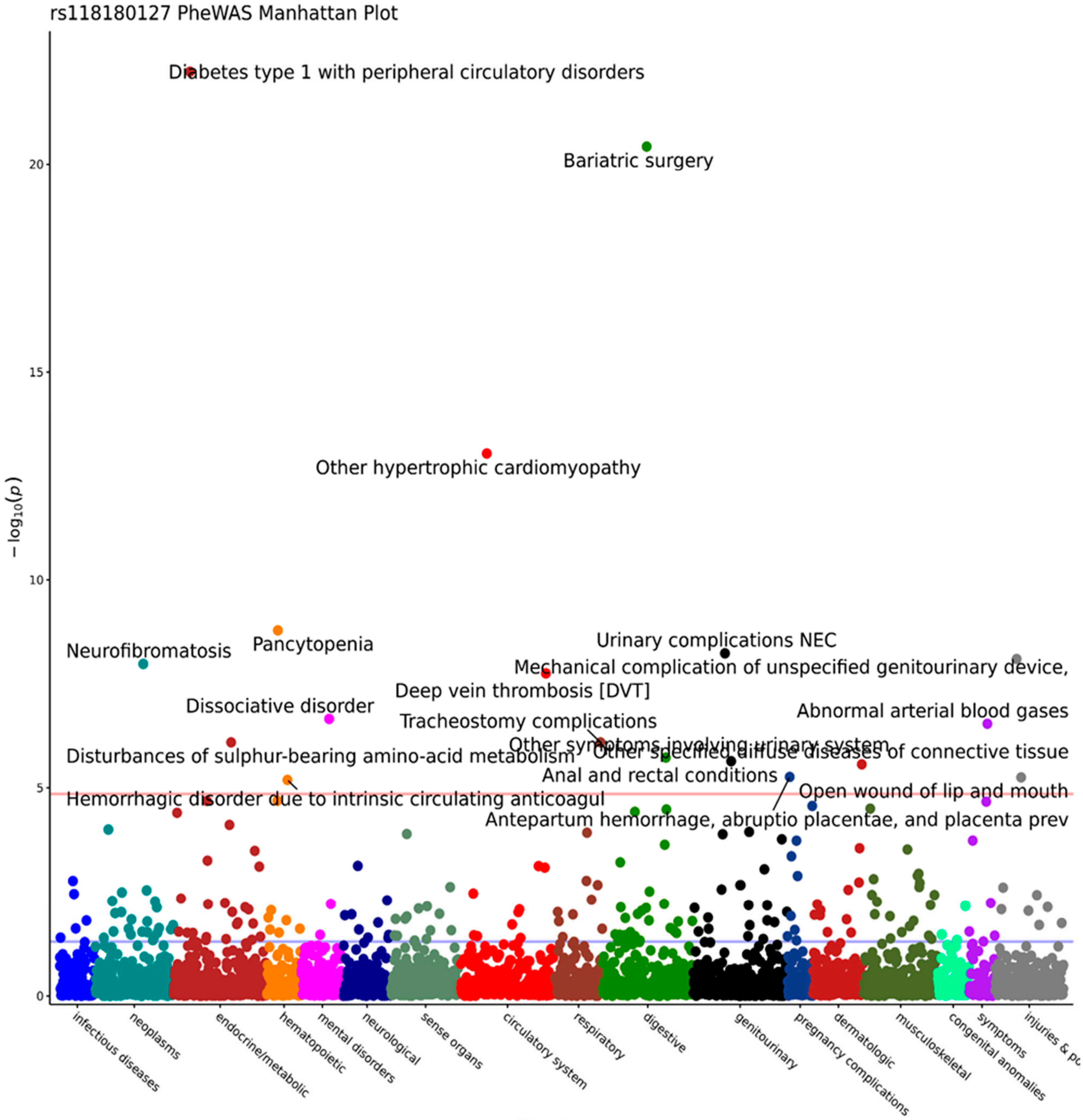
rs118180127 PheWAS Manhattan Plot
Diabetes type 1 with peripheral circulatory disorders
Bariatric surgery
Other hypertrophic cardiomyopathy
Neurofibromatosis
Pancytopenia
Urinary complications NEC
Mechanical complication of unspecified genitourinary device,
Deep vein thrombosis [DVT]
Dissociative disorder
Abnormal arterial blood gases
Tracheostomy complications
Other symptoms involving urinary system
Disturbances of sulphur-bearing amino-acid metabolism
Other specified diffuse diseases of connective tissue
Anal and rectal conditions
Open wound of lip and mouth
Hemorrhagic disorder due to intrinsic circulating anticoagul
Antepartum hemorrhage, abruptio placentae, and placenta prev
$-\log_{10}(p)$
0
5
10
15
20
infectious diseases
neoplasms
endocrine/metabolic
hematopoietic
mental disorders
neurological
sense organs
circulatory system
respiratory
digestive
genitourinary
pregnancy complications
dermatologic
musculoskeletal
congenital anomalies
symptoms
Phenotypes

(F)

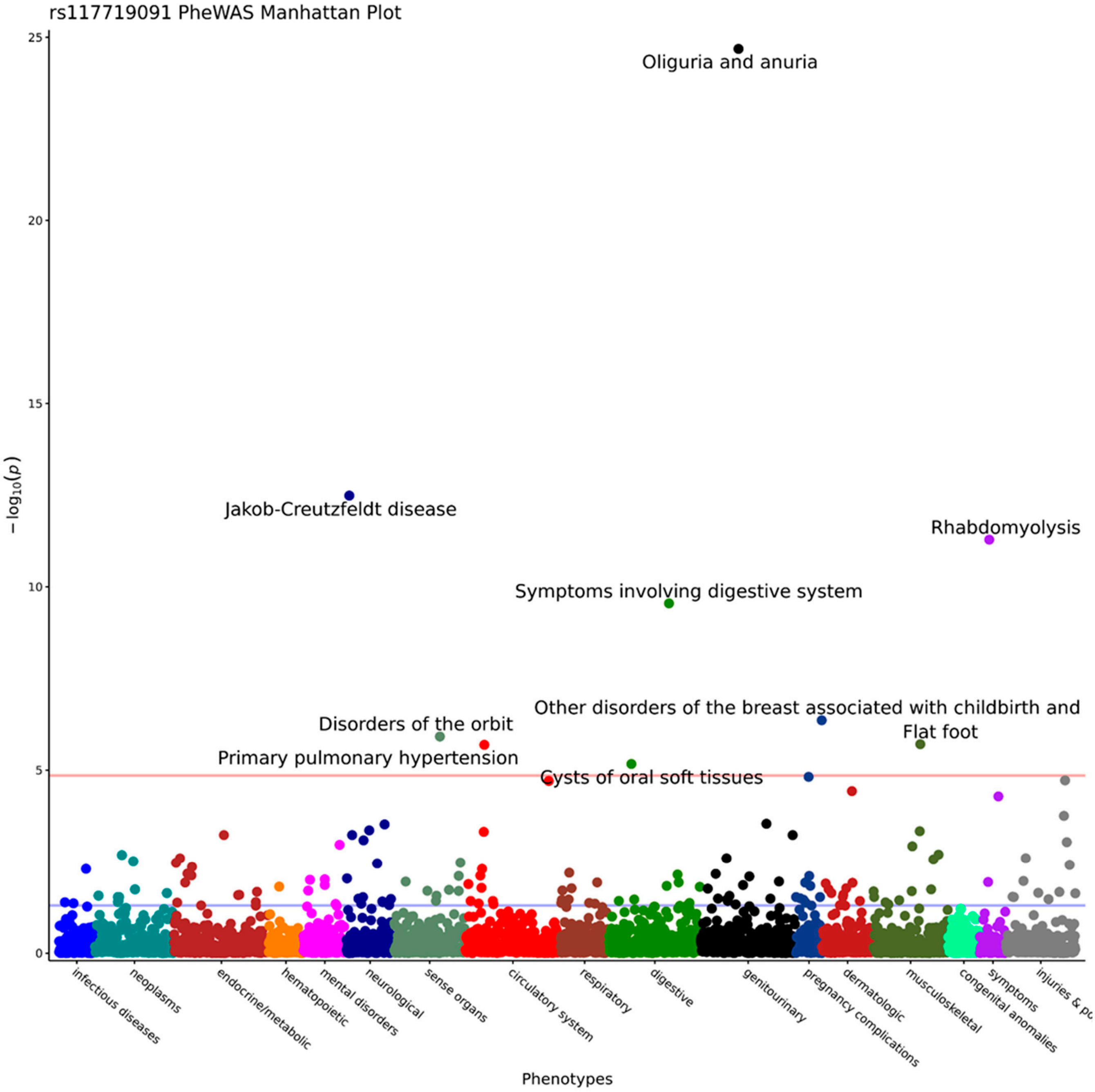
rs117719091 PheWAS Manhattan Plot
25
20
15
10
5
0
− log₁₀(p)
Oliguria and anuria
Jakob-Creutzfeldt disease
Rhabdomyolysis
Symptoms involving digestive system
Other disorders of the breast associated with childbirth and
Flat foot
Disorders of the orbit
Primary pulmonary hypertension
Cysts of oral soft tissues
infectious diseases
neoplasms
endocrine/metabolic
hematopoietic
mental disorders
neurological
sense organs
circulatory system
respiratory
digestive
genitourinary
pregnancy complications
dermatologic
musculoskeletal
congenital anomalies
symptoms
injuries & po
Phenotypes

(G)

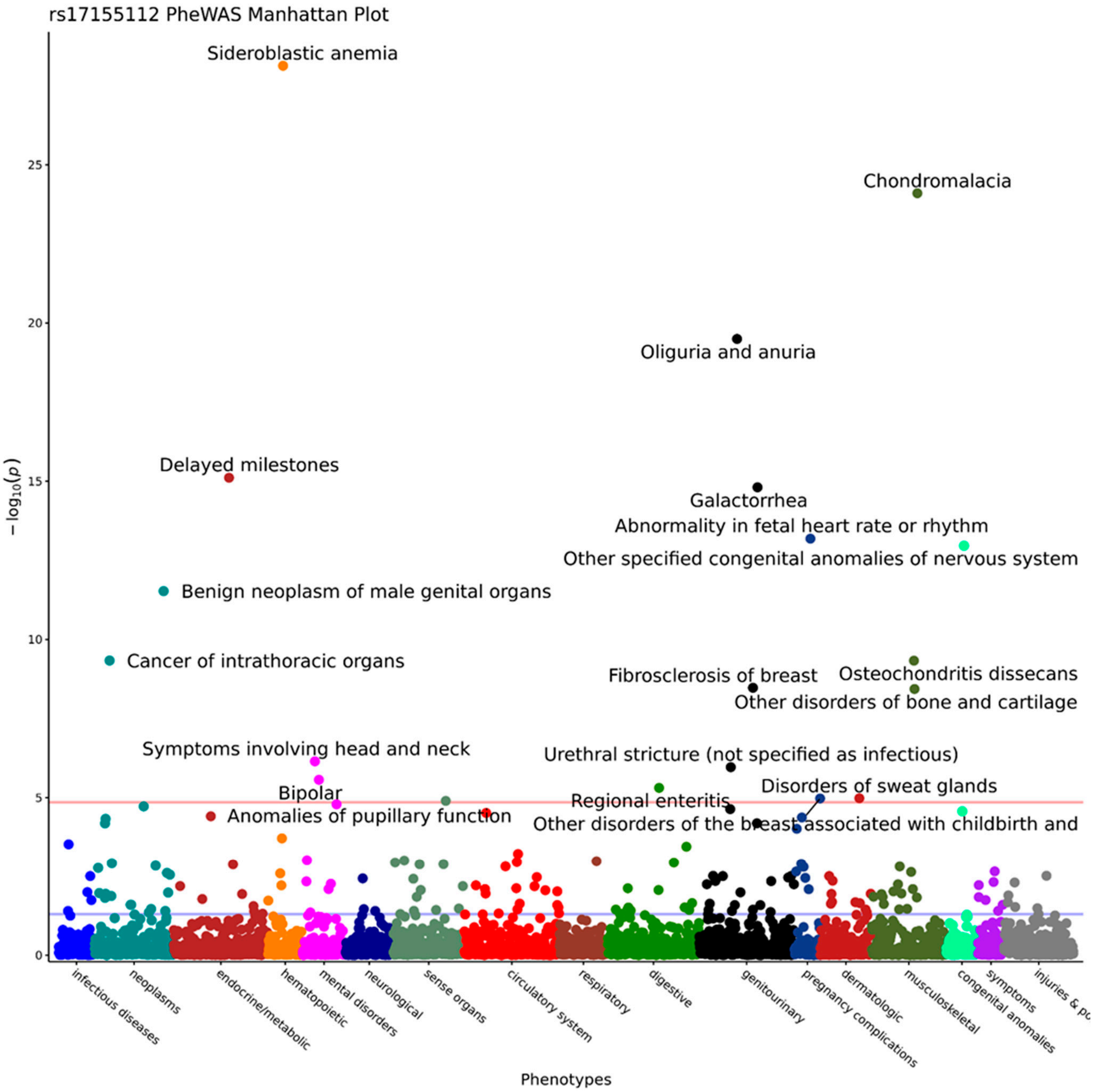
rs17155112 PheWAS Manhattan Plot
Sideroblastic anemia
Chondromalacia
Oliguria and anuria
Delayed milestones
Galactorrhea
Abnormality in fetal heart rate or rhythm
Other specified congenital anomalies of nervous system
Benign neoplasm of male genital organs
Cancer of intrathoracic organs
Fibrosclerosis of breast
Osteochondritis dissecans
Other disorders of bone and cartilage
Symptoms involving head and neck
Urethral stricture (not specified as infectious)
Bipolar
Disorders of sweat glands
Regional enteritis
Anomalies of pupillary function
Other disorders of the breast associated with childbirth and
$-\log_{10}(p)$
25
20
15
10
5
0
infectious diseases
neoplasms
endocrine/metabolic
hematopoietic
mental disorders
neurological
sense organs
circulatory system
respiratory
digestive
genitourinary
pregnancy complications
dermatologic
musculoskeletal
congenital anomalies
symptoms
Phenotypes

(H)

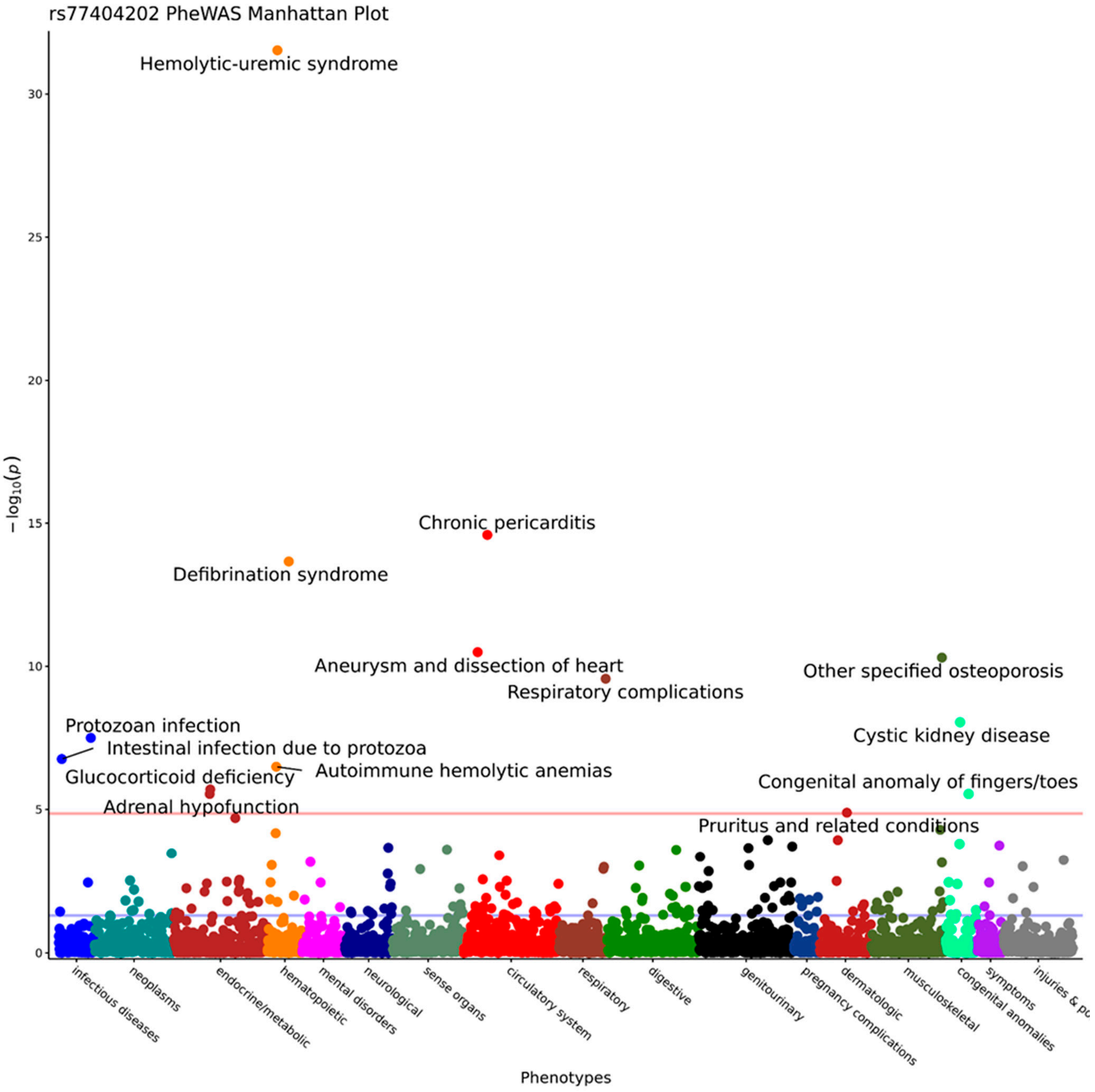

rs77404202 PheWAS Manhattan Plot
Hemolytic-uremic syndrome
Chronic pericarditis
Defibrination syndrome
Aneurysm and dissection of heart
Respiratory complications
Other specified osteoporosis
Protozoan infection
Intestinal infection due to protozoa
Glucocorticoid deficiency
Autoimmune hemolytic anemias
Adrenal hypofunction
Cystic kidney disease
Congenital anomaly of fingers/toes
Pruritus and related conditions
$-\log_{10}(p)$
0
5
10
15
20
25
30
infectious diseases
neoplasms
endocrine/metabolic
hematopoietic
mental disorders
neurological
sense organs
circulatory system
respiratory
digestive
genitourinary
pregnancy complications
dermatologic
musculoskeletal
congenital anomalies
symptoms
Phenotypes

(I)

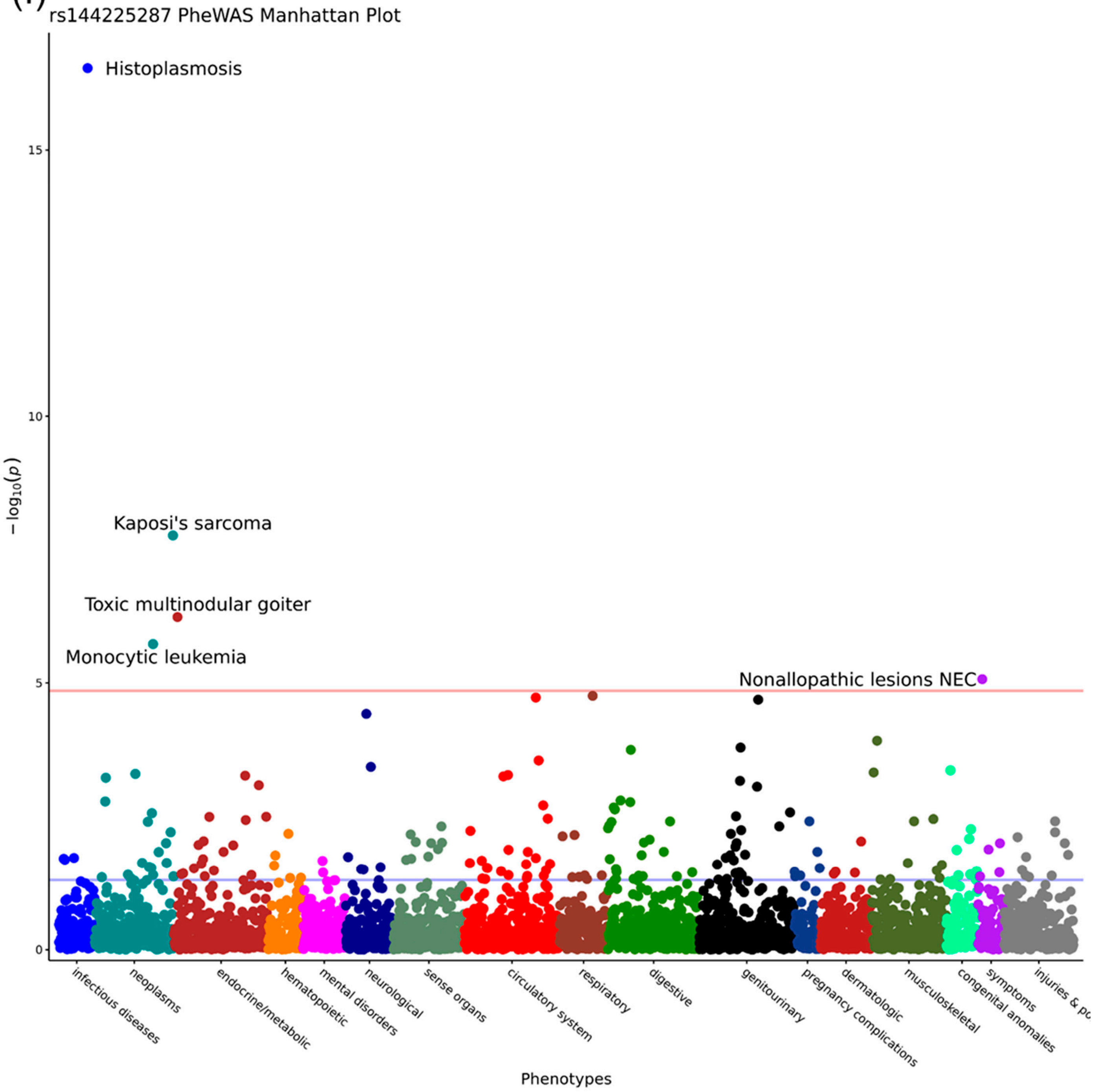

rs144225287 PheWAS Manhattan Plot
Histoplasmosis
Kaposi's sarcoma
Toxic multinodular goiter
Monocytic leukemia
Nonallopathic lesions NEC
15
10
5
0
− log10(p)
infectious diseases
neoplasms
endocrine/metabolic
hematopoietic
mental disorders
neurological
sense organs
circulatory system
respiratory
digestive
genitourinary
pregnancy complications
dermatologic
musculoskeletal
congenital anomalies
symptoms
Phenotypes

(J)

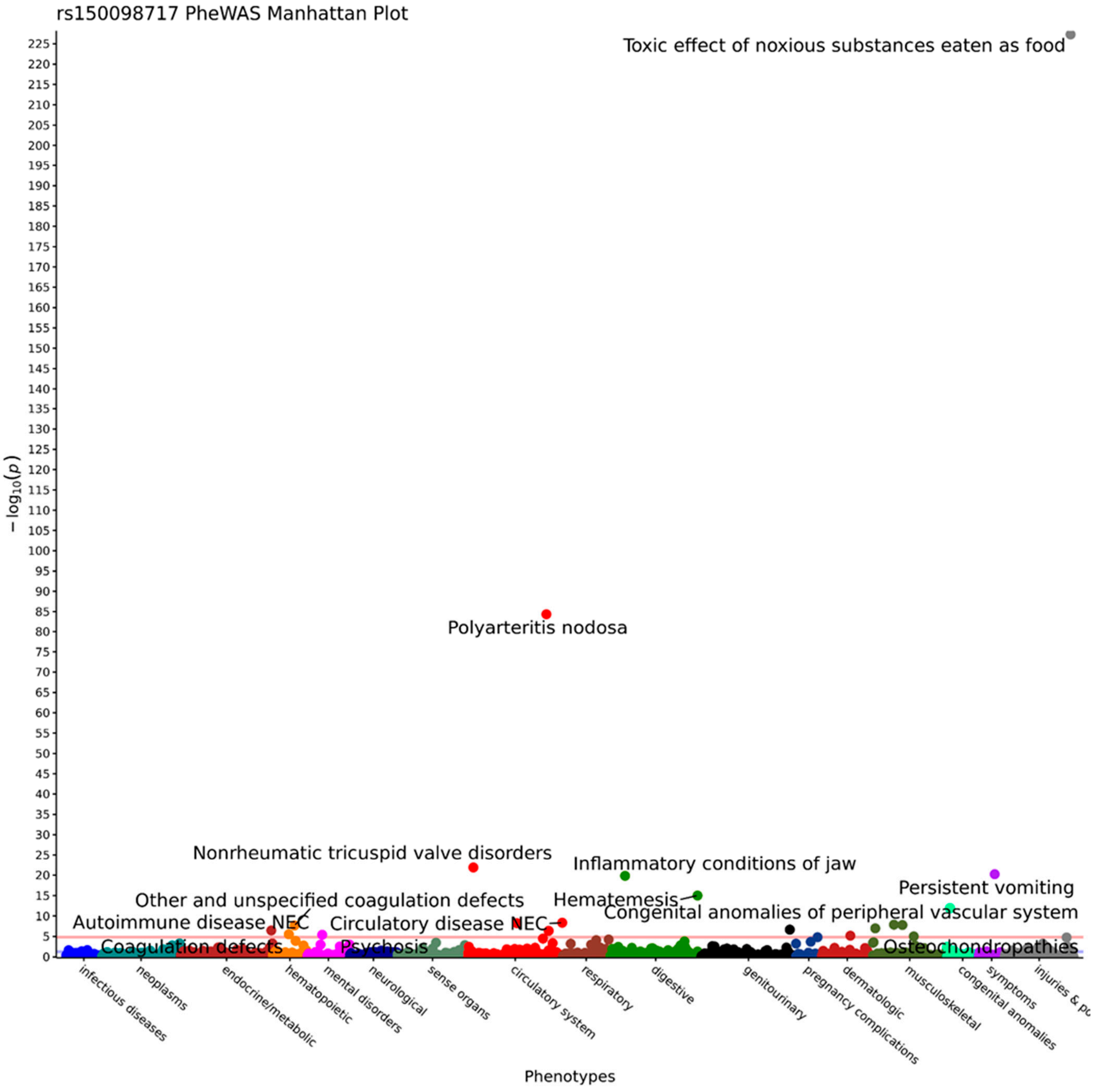
rs150098717 PheWAS Manhattan Plot
Toxic effect of noxious substances eaten as food
Polyarteritis nodosa
Nonrheumatic tricuspid valve disorders
Inflammatory conditions of jaw
Persistent vomiting
Other and unspecified coagulation defects
Hematemesis
Congenital anomalies of peripheral vascular system
Autoimmune disease NEC
Circulatory disease NEC
Coagulation defects
Psychosis
Osteochondropathies
$-\log_{10}(p)$
infectious diseases
neoplasms
endocrine/metabolic
hematopoietic
mental disorders
neurological
sense organs
circulatory system
respiratory
digestive
genitourinary
pregnancy complications
dermatologic
musculoskeletal
congenital anomalies
symptoms
Phenotypes

(K)

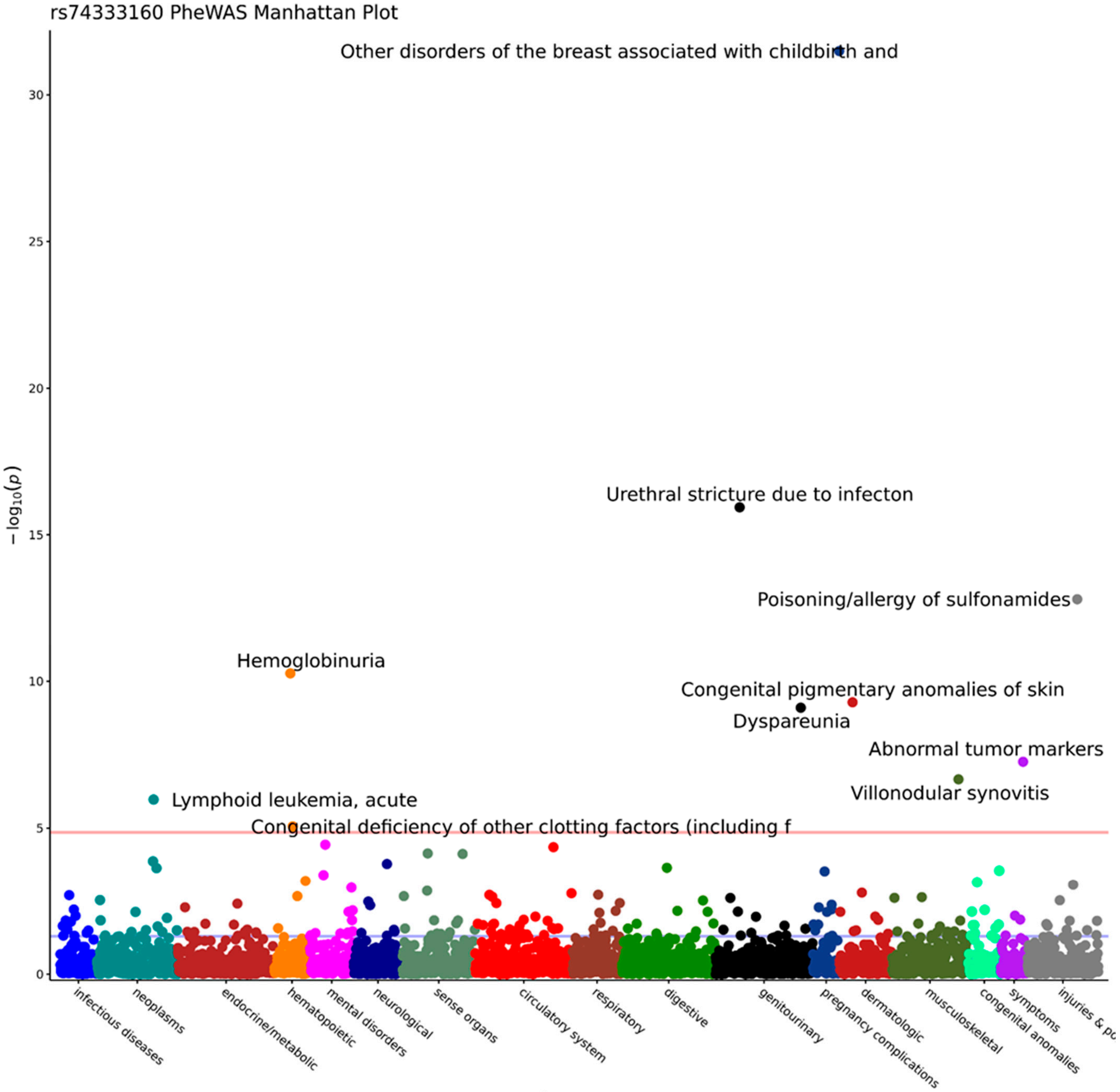
rs74333160 PheWAS Manhattan Plot
Other disorders of the breast associated with childbirth and
Urethral stricture due to infecton
Poisoning/allergy of sulfonamides
Hemoglobinuria
Congenital pigmentary anomalies of skin
Dyspareunia
Abnormal tumor markers
Villonodular synovitis
Lymphoid leukemia, acute
Congenital deficiency of other clotting factors (including f
$-\log_{10}(p)$
0
5
10
15
20
25
30
infectious diseases
neoplasms
endocrine/metabolic
hematopoietic
mental disorders
neurological
sense organs
circulatory system
respiratory
digestive
genitourinary
pregnancy complications
dermatologic
musculoskeletal
congenital anomalies
symptoms
injuries & po
Phenotypes

(L)

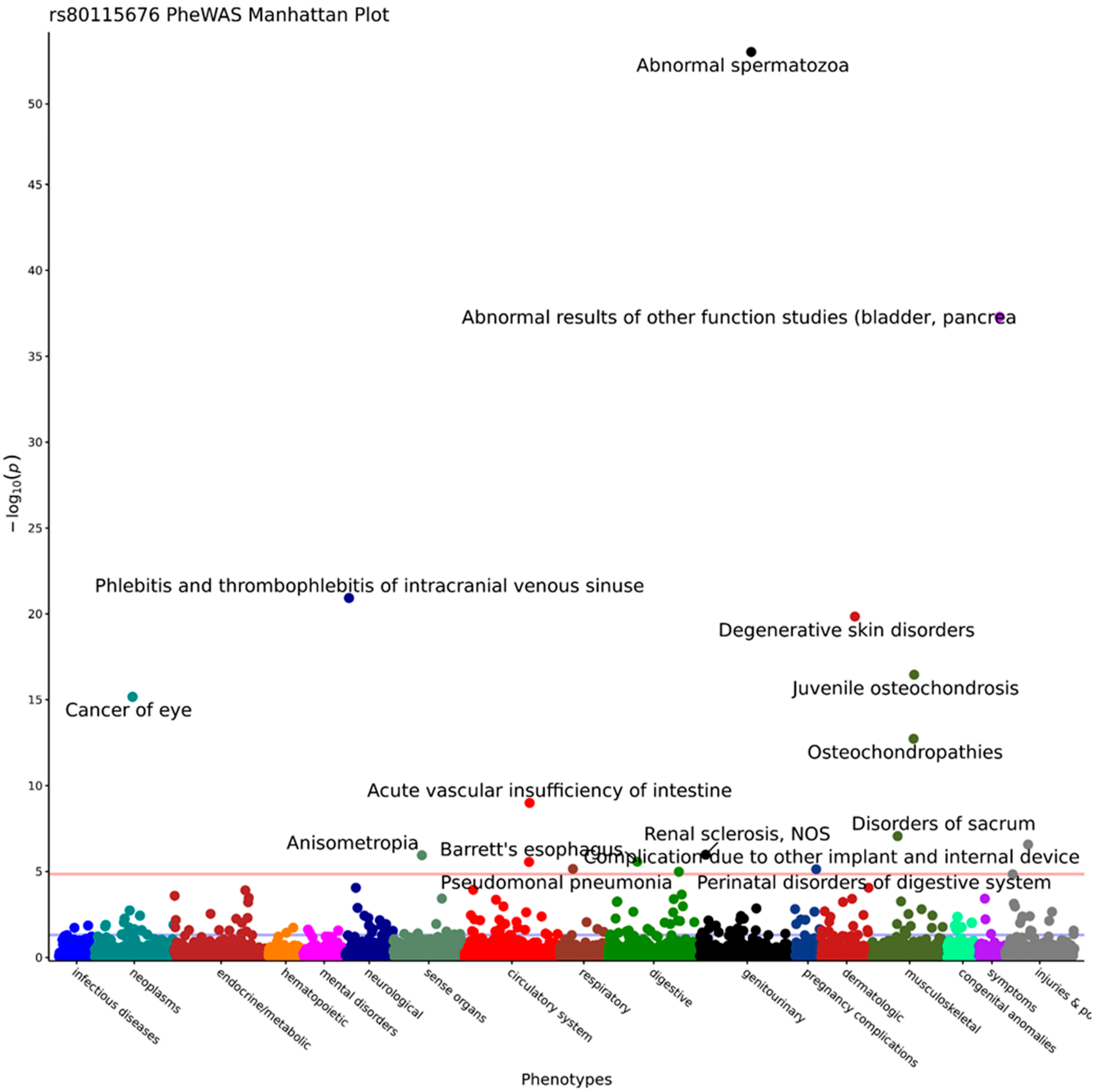

rs80115676 PheWAS Manhattan Plot
Abnormal spermatozoa
Abnormal results of other function studies (bladder, pancrea
Phlebitis and thrombophlebitis of intracranial venous sinuse
Degenerative skin disorders
Juvenile osteochondrosis
Cancer of eye
Osteochondropathies
Acute vascular insufficiency of intestine
Disorders of sacrum
Renal sclerosis, NOS
Anisometropia
Barrett's esophagus
Complication due to other implant and internal device
Pseudomonal pneumonia
Perinatal disorders of digestive system
$-\log_{10}(p)$
0
5
10
15
20
25
30
35
40
45
50
infectious diseases
neoplasms
endocrine/metabolic
hematopoietic
mental disorders
neurological
sense organs
circulatory system
respiratory
digestive
genitourinary
pregnancy complications
dermatologic
musculoskeletal
congenital anomalies
symptoms
injuries & po
Phenotypes

(M)

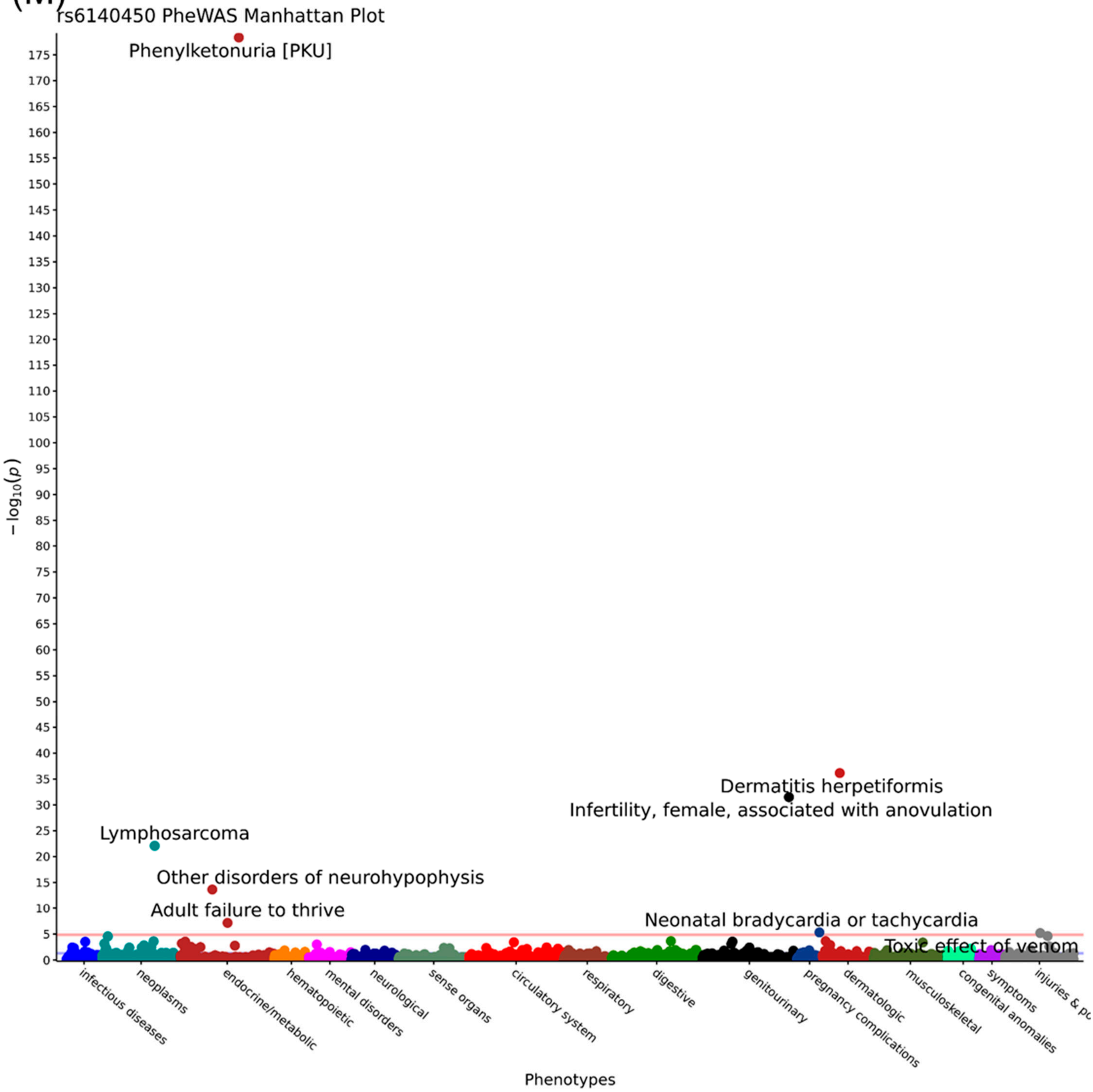
rs6140450 PheWAS Manhattan Plot
Phenylketonuria [PKU]
Dermatitis herpetiformis
Infertility, female, associated with anovulation
Lymphosarcoma
Other disorders of neurohypophysis
Adult failure to thrive
Neonatal bradycardia or tachycardia
Toxic effect of venom
$-\log_{10}(p)$
0
5
10
15
20
25
30
35
40
45
50
55
60
65
70
75
80
85
90
95
100
105
110
115
120
125
130
135
140
145
150
155
160
165
170
175
infectious diseases
neoplasms
endocrine/metabolic
hematopoietic
mental disorders
neurological
sense organs
circulatory system
respiratory
digestive
genitourinary
pregnancy complications
dermatologic
musculoskeletal
congenital anomalies
symptoms
Phenotypes

Figure S4. Expression of five novel loci-related 6 genes and survival in other cohorts. The related genes that exhibited different expression in tumor and adjacent normal tissues were validated in our study by using the TCGA-LIHC dataset. The red box depicts the tumor tissue, and the green box depicts the normal tissue. Survival analysis of middle and bottom panels were conducted from TCGA-LIHC and GEO data, respectively. (A) *F11*. (B) *F11-AS1*. (C) *FRMD4A*. (D) *KIAA0232*. (D) *LINC02511*. (E) *PFKFB3*.

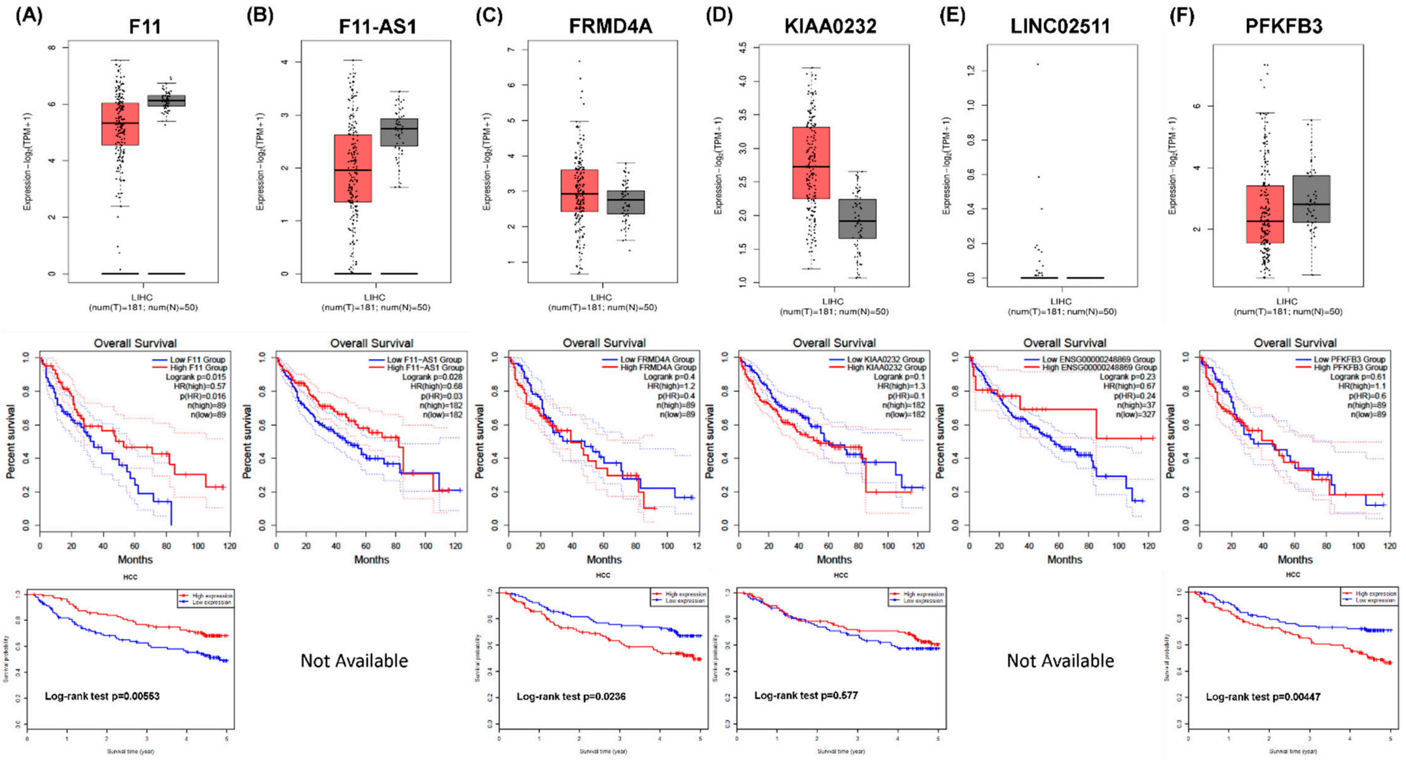

Figure S5. Results of the analysis of the expression and somatic mutations of 13 novel loci-related genes by using the TCGA-LIHC cohort. (A) Results of the somatic mutations of 12 loci related genes. **(B)** Results of differential expression between HCC and noncancerous liver tissues.

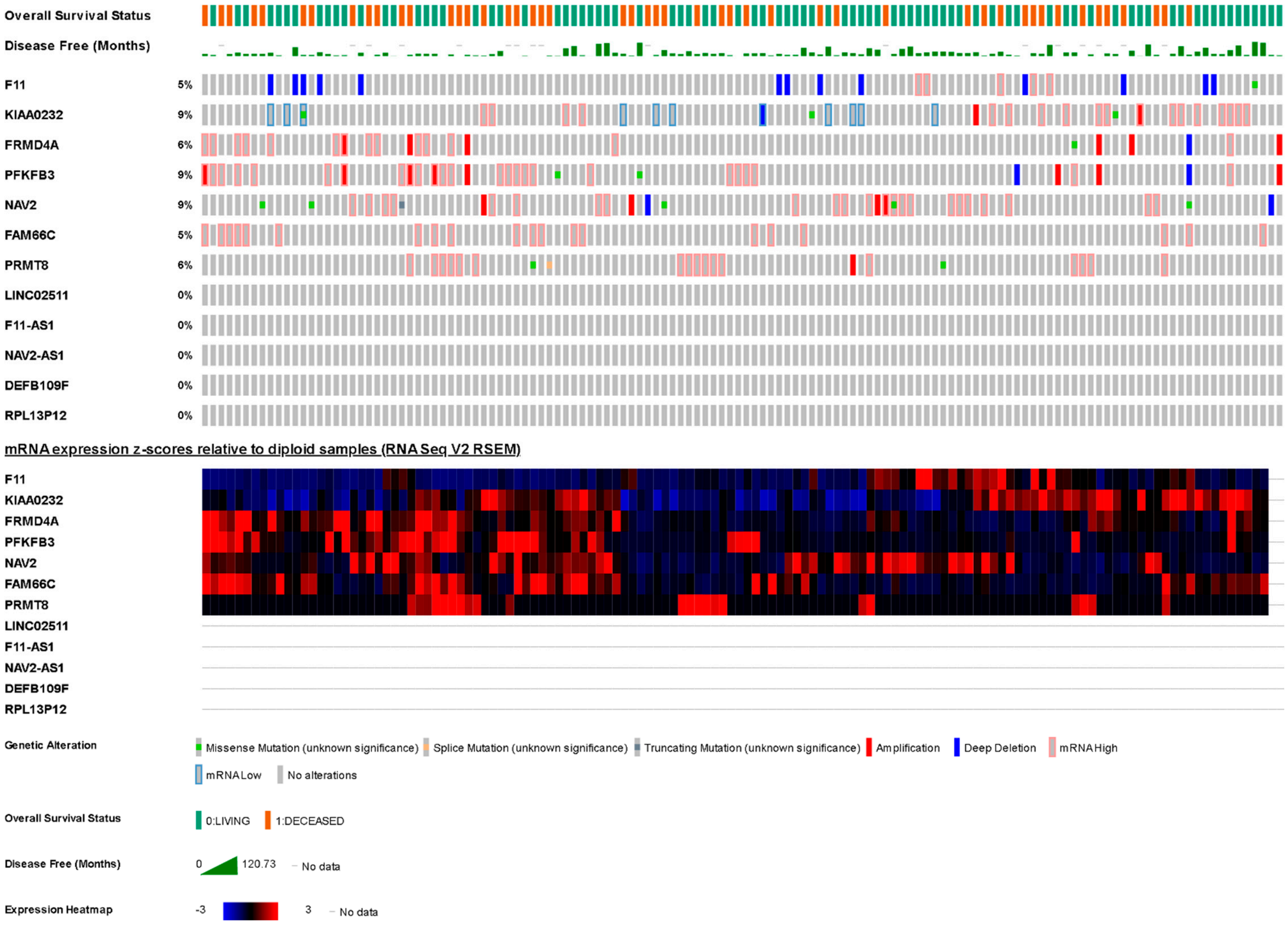

Figure S6. Results of the performance for top 5%, 10%, 15%, and 20% PRS only, clinical data only, or combined both to predict HCC risk. (A) ROC curve of top 5% of both extremes. **(B)** ROC curve of the top 10% of both extremes. **(C)** ROC curve of the top 15% of both extremes. **(D)** ROC curve of the top 20% of both extreme. Demographic factors (age, sex, and BMI) and bioclinical data (albumin, HBV surface antigen, and HCV antibody) were used in model building. Combine means both the PRS and clinical data were used in model building.

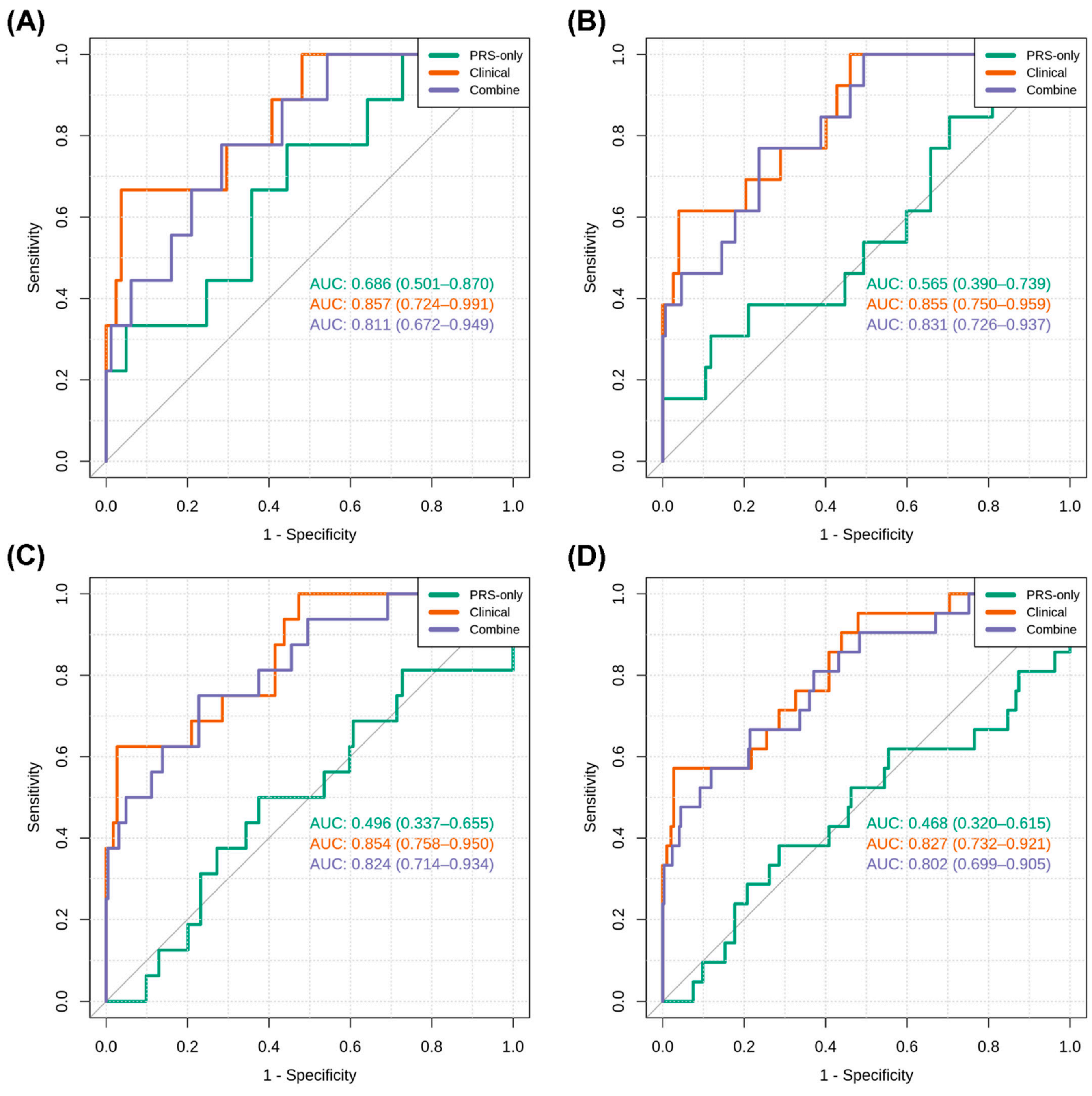

Figure S7. PheWAS Manhattan plot for HCC PRS in Taiwanese with HCC

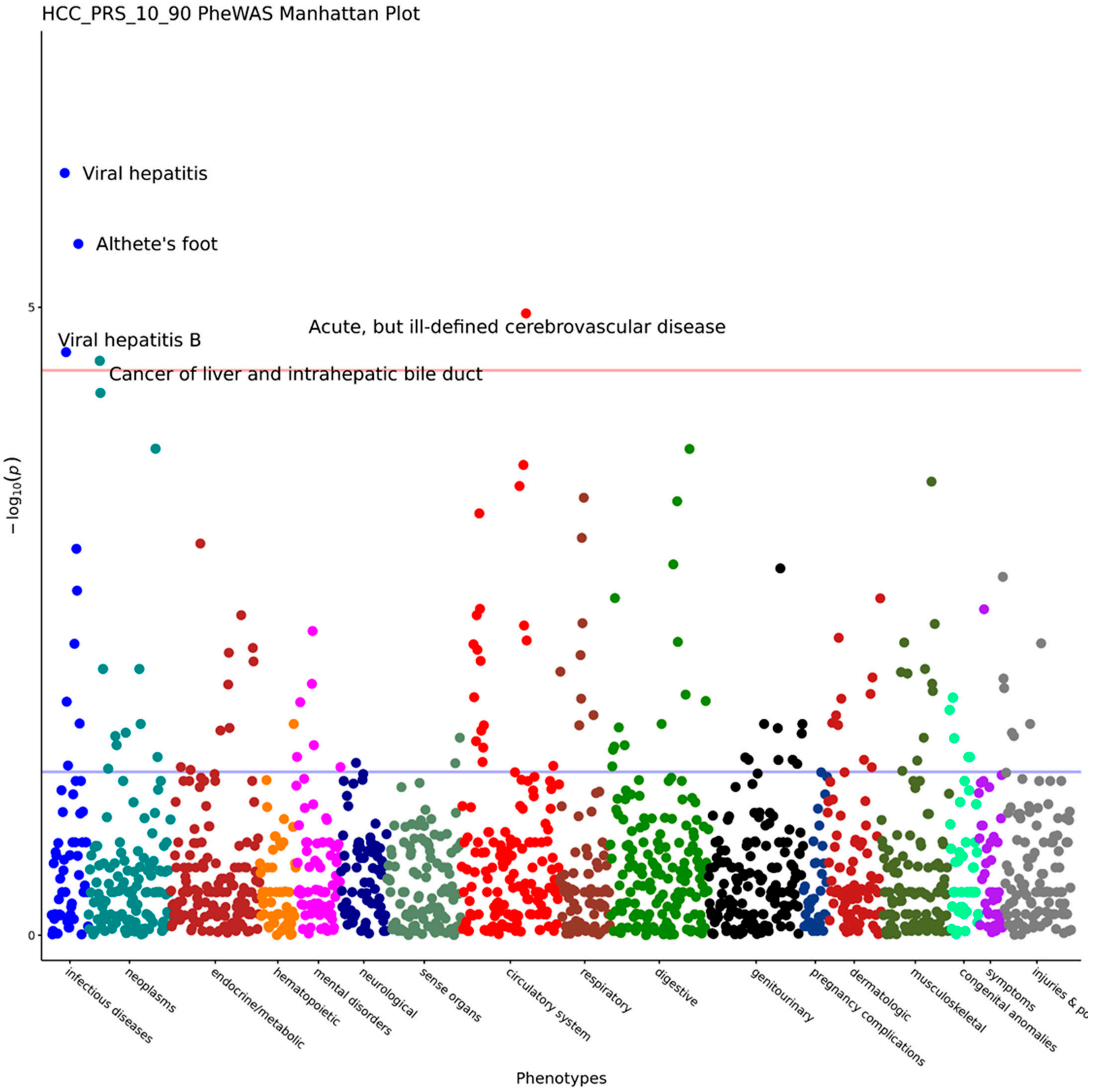

Table S1. Variants were significant in our discovery GWAS but not in other studies, and the results of the meta-analysis by using the UK Biobank and BBJ cohort were shown. Abbreviations: Chr, chromosome; RA, reference allele; EA, effect allele; AF, allele frequency; PAF, publish allele frequency; OR, odds ratio; CI, confidence interval; MA, meta-analysis; MAF, minor allele frequency.

This table is shown in Supplementary_Table_S1.xlsx

Table S2. Variants were not significant in our discovery GWAS but significant after performing a meta-analysis by using data from the UK Biobank and BBJ cohort. Abbreviations: Chr, chromosome; RA, reference allele; EA, effect allele; AF, allele frequency; PAF, publish allele frequency; OR, odds ratio; CI, confidence interval; MA, meta-analysis; MAF, minor allele frequency.

The table is shown in Supplementary_Table_S2.xlsx

Table S3. Variants were significant in our study and other previous studies. Abbreviations: Chr, chromosome; RA, reference allele; EA, effect allele; AF, allele frequency; PAF, publish allele frequency; OR, odds ratio; CI, confidence interval; MA, meta-analysis; MAF, minor allele frequency.

The table is shown in Supplementary_Table_S3.xlsx

Table S4. Summary statistics of 13 novel SNPs in another independent cohort in Taiwanese

CHR	SNP	BP	A1	F_A	F_U	A2	P	OR	SE	L95	U95	MAF	NCHROBS
1	rs187199523	1.94E+08	T	0.02054	0.01785	A	0.3548	1.154	0.1646	0.8359	1.593	0.01804	602952
4	rs144285059	1.37E+08	GA	0.02965	0.02065	G	0.01009	1.449	0.1415	1.098	1.912	0.02124	574682
4	rs148610742	1.86E+08	T	0.01482	0.01164	C	0.2077	1.277	0.1947	0.8716	1.87	0.01154	594484
8	rs118180127	8513430	A	0.05118	0.05448	T	0.5319	0.9361	0.103	0.765	1.146	0.05502	637032
10	rs117719091	6227313	T	0.03941	0.04448	C	0.2829	0.8812	0.1166	0.7011	1.108	0.04397	637032
10	rs17155112	14357172	A	0.01226	0.01164	G	0.7054	1.054	0.2106	0.6975	1.592	0.01165	614518
11	rs77404202	20117743	T	0.04964	0.04412	C	0.2349	1.132	0.1046	0.922	1.389	0.04336	637032
12	rs150098717	8198462	T	0.04299	0.03885	C	0.3326	1.111	0.112	0.8923	1.384	0.03815	637032
14	rs74333160	1.01E+08	G	0.06807	0.06215	T	0.2693	1.102	0.09016	0.9236	1.315	0.06193	637032
17	rs80115676	17375355	G	0.05169	0.05193	A	0.9796	0.995	0.1025	0.8139	1.216	0.05154	637032
4	rs140233124	6834347	-	NA									
12	rs144225287	3568611	-	NA									
20	rs6140450	7873320	C	NA									

Table S5. PheWAS Results in Taiwanese with HCC. (A) rs187199523. (B) rs140233124. (C) rs144285059. (D) rs148610742. (E) rs118180127. (F) rs117719091. (G) rs17155112. (H) rs77404202. (I) rs144225287. (J) rs150098717. (K) rs74333160. (L) rs80115676. (M) rs61404501.

This table is shown in Supplementary_Table_S5.xlsx

Table S6.1. Differential gene expression of novel loci related genes between the noncancerous and cancerous tissues of Taiwanese patients with HCC. The 16 novel loci-related genes were retrieved; 9 genes are shown in the table below, and the other 7 genes were removed because their alignment read count was too low. Abbreviations: logFC, log fold change; logCPM, log of count per million.

ENSGID	*Gene*	*logFC*	*logCPM*	*F*	*PValue*	*FDR*
ENSG00000170525	PFKFB3	-1.7038	5.82257	53.8401	4.46E-11	1.06E-09
ENSG00000170871	KIAA0232	0.78244	6.30025	44.2176	1.28E-09	1.85E-08
ENSG00000248869	LINC02511	3.5091	-2.541	15.6986	1.34E-04	4.44E-04
ENSG00000088926	F11	-0.7353	6.61583	10.4003	1.67E-03	4.14E-03
ENSG00000151474	FRMD4A	-0.4199	4.95127	6.89554	9.91E-03	2.00E-02
ENSG00000166833	NAV2	-0.3756	7.32916	4.20797	4.27E-02	7.15E-02
ENSG00000227240	AL136456.1	0.35129	1.36369	0.71478	4.00E-01	4.84E-01
ENSG00000226711	FAM66C	0.19011	-0.9222	0.38345	5.37E-01	6.15E-01
ENSG00000215030	RPL13P12	-0.1638	1.17802	0.37282	5.43E-01	6.21E-01

Table S6.2. Survival analyses of 13 novel loci-related genes. We used a threshold of 75 for mRNA expression to group those with high and low expression. Abbreviation: NA, not available.

ENSGID	*Gene*	*G1_N*	*G2_N*	*Log Rank P-val*
ENSG00000088926	F11	38	37	6.93E-01
ENSG00000111218	PRMT8*	61	14	2.21E-01
ENSG00000151474	FRMD4A	38	37	6.58E-01
ENSG00000166833	NAV2	38	37	6.14E-01
ENSG00000170525	PFKFB3	38	37	7.40E-01
ENSG00000170871	KIAA0232	38	37	6.88E-01
ENSG00000215030	RPL13P12	38	37	6.30E-01
ENSG00000226711	FAM66C	38	37	6.18E-01
ENSG00000227240	AL136456.1	38	37	1.62E-01
ENSG00000244050	DEFB109F*	74	1	7.68E-01
ENSG00000248869	LINC02511*	62	13	8.81E-01
ENSG00000251165	F11-AS1*	48	27	3.43E-02
ENSG00000253343	AC114550.2*	66	9	3.95E-01
ENSG00000254894	NAV2-AS1*	68	7	4.76E-01
ENSG00000277315	AL021879.1*	74	1	8.68E-01
ENSG00000288245	AL355836.4*	75	0	NA

*We grouped those expressing or not expressing the gene because most of the cases did not express this gene.

Table S7.1. Results of HLA-A subtypes significantly associated with HCC in the Taiwanese population. Abbreviations: OR, odds ratio; CI, confidence interval; N, allele number; NA, not available.

Genotype	*Control*	*Case*	*P-value*[a]	*Q-value*[b]	*OR*	*95% CI*	*Allele Frequency*
	N=241,306	*N=5,858*					
*01:01	1071 (0.44%)	23 (0.39%)	6.20E-01	1.42E+00	1.15	0.76-1.83	0.58%
*02:01	8574 (3.55%)	217 (3.7%)	6.94E-01	1.01E+00	0.97	0.85-1.12	9.94%
*02:03	5734 (2.38%)	158 (2.7%)	1.66E-01	2.66E+00	0.89	0.76-1.06	6.21%
*02:05	66 (0.03%)	2 (0.03%)	6.80E-01	1.21E+00	0.81	0.22-6.87	0.05%
*02:06	362 (0.15%)	9 (0.15%)	8.66E-01	1.15E+00	0.99	0.52-2.19	2.70%
*02:07	18942 (7.85%)	459 (7.84%)	6.91E-01	1.11E+00	1.02	0.93-1.13	10.72%
*03:01	682 (0.28%)	13 (0.22%)	4.55E-01	1.82E+00	1.3	0.75-2.45	0.39%
*11:01	54869 (22.74%)	1333 (22.76%)	4.80E-01	1.54E+00	1.02	0.96-1.1	28.82%
*11:02	8801 (3.65%)	203 (3.47%)	3.40E-01	1.82E+00	1.07	0.93-1.25	4.98%
*24:02	32348 (13.41%)	815 (13.91%)	5.23E-01	1.39E+00	0.98	0.9-1.06	17.31%
*26:01	4820 (2%)	119 (2.03%)	1.00E+00	1.14E+00	1	0.83-1.21	2.79%
*30:01	2690 (1.11%)	66 (1.13%)	1.00E+00	1.14E+00	1.01	0.79-1.31	1.30%
*31:01	3599 (1.49%)	89 (1.52%)	9.57E-01	1.18E+00	1	0.81-1.25	2.00%
*32:01	642 (0.27%)	21 (0.36%)	2.03E-01	1.62E+00	0.75	0.49-1.23	0.37%
*33:03	22584 (9.36%)	567 (9.68%)	6.78E-01	1.36E+00	0.98	0.9-1.07	11.06%
*68:01	30 (0.01%)	0 (0%)	NA	NA	NA	NA	0.15%

[a]*P* value for Fisher's exact test.
[b]Q value for FDR-BH adjustment.

Table S7.2. Results of HLA-A subtypes significantly associated with HBV infection in the Taiwanese population. Abbreviations: OR, odds ratio; CI, confidence interval; N, allele number; NA, not available.

Genotype	*HBSag-*	*HBSag+*	*P-value*[a]	*Q-value*[b]	*OR*	*95% CI*	*Allele Frequency*
	N=114,898	*N=22,632*					
*01:01	488 (0.42%)	77 (0.34%)	4.78E-02	1.28E-01	1.28	1-1.65	0.58%
*02:01	4130 (3.59%)	859 (3.8%)	3.60E-01	4.43E-01	0.97	0.9-1.04	9.94%
*02:03	2840 (2.47%)	603 (2.66%)	2.27E-01	3.63E-01	0.95	0.86-1.04	6.21%
*02:05	36 (0.03%)	4 (0.02%)	3.94E-01	4.50E-01	1.81	0.65-7.01	0.05%
*02:06	181 (0.16%)	48 (0.21%)	9.22E-02	1.84E-01	0.76	0.55-1.07	2.70%
*02:07	9037 (7.87%)	1863 (8.23%)	3.13E-01	4.17E-01	0.97	0.92-1.03	10.72%
*03:01	322 (0.28%)	57 (0.25%)	4.08E-01	4.08E-01	1.14	0.86-1.54	0.39%
*11:01	25957 (22.59%)	5055 (22.34%)	9.69E-03	3.10E-02	1.05	1.01-1.09	28.82%
*11:02	4213 (3.67%)	821 (3.63%)	4.04E-01	4.31E-01	1.03	0.96-1.12	4.98%
*24:02	15116 (13.16%)	3334 (14.73%)	1.12E-07	1.78E-06	0.89	0.85-0.93	17.31%
*26:01	2244 (1.95%)	495 (2.19%)	6.16E-02	1.41E-01	0.91	0.82-1.01	2.79%
*30:01	1319 (1.15%)	178 (0.79%)	1.56E-07	1.25E-06	1.5	1.28-1.76	1.30%
*31:01	1724 (1.5%)	378 (1.67%)	1.32E-01	2.35E-01	0.92	0.82-1.03	2.00%
*32:01	325 (0.28%)	41 (0.18%)	3.92E-03	1.57E-02	1.6	1.15-2.27	0.37%
*33:03	10922 (9.51%)	2055 (9.08%)	2.60E-03	1.39E-02	1.08	1.03-1.14	11.06%

*68:01	12 (0.01%)	0 (0%)	NA	NA	NA	NA	0.15%

[a]*P* value for Fisher's exact test.
[b]Q value for FDR-BH adjustment.

Table S7.3. Results of HLA-A subtypes significantly associated with HCV infection in the Taiwanese population. Abbreviations: OR, odds ratio; CI, confidence interval; N, allele number.

Genotype	*HCVab-*	*HCVab+*	*P-value*[a]	*Q-value*[b]	*OR*	*95% CI*	*Allele Frequency*
	N=113,974	*N=10,048*					
*01:01	488 (0.43%)	34 (0.34%)	1.74E-01	5.56E-01	1.28	0.91-1.88	0.58%
*02:01	4135 (3.63%)	372 (3.7%)	8.89E-01	9.48E-01	0.99	0.89-1.11	9.94%
*02:03	2795 (2.45%)	254 (2.53%)	7.88E-01	1.05E+00	0.98	0.86-1.12	6.21%
*02:05	34 (0.03%)	2 (0.02%)	7.66E-01	1.11E+00	1.52	0.39-13.05	0.05%
*02:06	189 (0.17%)	15 (0.15%)	7.98E-01	9.82E-01	1.13	0.66-2.05	2.70%
*02:07	9100 (7.98%)	759 (7.55%)	5.58E-02	4.47E-01	1.08	1-1.17	10.72%
*03:01	309 (0.27%)	19 (0.19%)	1.29E-01	5.18E-01	1.45	0.91-2.45	0.39%
*11:01	25694 (22.54%)	2325 (23.14%)	4.65E-01	1.24E+00	0.98	0.93-1.03	28.82%
*11:02	4270 (3.75%)	346 (3.44%)	7.31E-02	3.90E-01	1.11	0.99-1.24	4.98%
*24:02	15295 (13.42%)	1369 (13.62%)	9.37E-01	9.37E-01	1	0.94-1.06	17.31%
*26:01	2271 (1.99%)	199 (1.98%)	8.23E-01	9.41E-01	1.02	0.88-1.19	2.79%
*30:01	1247 (1.09%)	115 (1.14%)	7.27E-01	1.16E+00	0.97	0.8-1.18	1.30%
*31:01	1754 (1.54%)	150 (1.49%)	6.42E-01	1.14E+00	1.05	0.88-1.25	2.00%
*32:01	298 (0.26%)	23 (0.23%)	6.10E-01	1.22E+00	1.16	0.76-1.86	0.37%
*33:03	10686 (9.38%)	1035 (10.3%)	7.73E-03	1.24E-01	0.91	0.85-0.98	11.06%
*68:01	9 (0.01%)	1 (0.01%)	5.75E-01	1.31E+00	0.8	0.11-35.24	0.15%

[a]*P* value for Fisher's exact test.
[b]Q-value for FDR-BH adjustment.

Table S7.4. Results of HLA-B subtypes significantly associated with HCC in the Taiwanese population. Abbreviations: OR, odds ratio; CI, confidence interval; N, allele number; NA, not available.

Genotype	*Control*	*Case*	*P-value*[a]	*Q-value*[b]	*OR*	*95% CI*	*Allele Frequency*
	N=241,306	*N=5,858*					
*07:02	437 (0.18%)	13 (0.22%)	4.39E-01	9.05E-01	0.82	0.47-1.56	0.35%
*13:01	11011 (4.56%)	282 (4.81%)	4.22E-01	9.29E-01	0.95	0.84-1.08	6.39%
*13:02	2686 (1.11%)	66 (1.13%)	9.50E-01	1.12E+00	0.99	0.78-1.29	1.44%
*15:01	645 (0.27%)	10 (0.17%)	1.97E-01	8.12E-01	1.58	0.85-3.31	4.12%
*15:02	7135 (2.96%)	178 (3.04%)	7.83E-01	1.08E+00	0.98	0.84-1.15	4.22%
*15:11	615 (0.25%)	16 (0.27%)	7.93E-01	1.05E+00	0.94	0.57-1.66	0.67%
*15:18	621 (0.26%)	17 (0.29%)	6.02E-01	9.46E-01	0.89	0.55-1.54	0.63%
*15:25	1230 (0.51%)	24 (0.41%)	3.07E-01	9.20E-01	1.26	0.84-1.97	0.76%
*27:04	5389 (2.23%)	119 (2.03%)	2.80E-01	9.24E-01	1.11	0.92-1.35	3.13%

*35:01	2801 (1.16%)	67 (1.14%)	9.51E-01	1.08E+00	1.02	0.8-1.33	2.78%
*35:03	152 (0.06%)	7 (0.12%)	1.09E-01	7.21E-01	0.53	0.25-1.34	0.12%
*37:01	637 (0.26%)	23 (0.39%)	7.21E-02	5.95E-01	0.68	0.45-1.08	0.37%
*38:02	6922 (2.87%)	206 (3.52%)	5.32E-03	8.79E-02	0.81	0.7-0.94	4.25%
*39:01	7 (0%)	0 (0%)	NA	NA	NA	NA	2.73%
*40:01	37737 (15.64%)	904 (15.43%)	4.97E-01	9.65E-01	1.03	0.95-1.11	20.96%
*40:02	2550 (1.06%)	55 (0.94%)	3.99E-01	1.01E+00	1.14	0.87-1.52	2.19%
*40:06	2231 (0.92%)	46 (0.79%)	2.68E-01	9.84E-01	1.19	0.89-1.63	1.27%
*44:02	222 (0.09%)	3 (0.05%)	3.85E-01	1.06E+00	1.81	0.61-8.85	0.21%
*44:03	479 (0.2%)	11 (0.19%)	1.00E+00	1.10E+00	1.06	0.59-2.15	0.39%
*46:01	23522 (9.75%)	580 (9.9%)	8.18E-01	1.04E+00	0.99	0.9-1.09	13.52%
*48:01	1057 (0.44%)	22 (0.38%)	5.47E-01	9.50E-01	1.18	0.77-1.89	1.48%
*50:01	103 (0.04%)	1 (0.02%)	5.24E-01	9.61E-01	2.52	0.44-100.46	0.09%
*51:01	5331 (2.21%)	108 (1.84%)	5.14E-02	5.65E-01	1.21	1-1.49	4.35%
*51:02	1343 (0.56%)	35 (0.6%)	6.58E-01	9.87E-01	0.94	0.67-1.35	1.24%
*52:01	1512 (0.63%)	42 (0.72%)	4.03E-01	9.49E-01	0.88	0.65-1.23	0.97%
*54:01	4815 (2%)	161 (2.75%)	1.27E-04	4.19E-03	0.72	0.61-0.85	3.91%
*55:02	1415 (0.59%)	33 (0.56%)	8.63E-01	1.05E+00	1.05	0.74-1.53	3.47%
*56:01	322 (0.13%)	12 (0.2%)	1.48E-01	6.99E-01	0.66	0.37-1.28	0.47%
*56:03	301 (0.12%)	5 (0.09%)	5.70E-01	9.41E-01	1.47	0.62-4.57	0.35%
*56:04	11 (0%)	0 (0%)	NA	NA	NA	NA	0.12%
*57:01	285 (0.12%)	5 (0.09%)	6.97E-01	1.00E+00	1.39	0.59-4.33	0.17%
*58:01	20121 (8.34%)	461 (7.87%)	1.40E-01	7.68E-01	1.08	0.98-1.19	10.77%
*67:01	1 (0%)	0 (0%)	NA	NA	NA	NA	0.24%

[a]*P* value for Fisher's exact test.
[b]Q-value for FDR-BH adjustment.

Table S7.5. Results of HLA-B subtypes significantly associated with HBV infection in the Taiwanese population. Abbreviations: OR, odds ratio; CI, confidence interval; N, allele number; NA, not available.

Genotype	*HBSag-*	*HBSag+*	*P-value*[a]	*Q-value*[b]	*OR*	*95% CI*	*Allele Frequency*
	N=114,898	*N=22,632*					
*07:02	208 (0.18%)	22 (0.1%)	2.53E-03	8.35E-03	1.9	1.22-3.1	0.35%
*13:01	5217 (4.54%)	1096 (4.84%)	1.66E-01	2.73E-01	0.95	0.89-1.02	6.39%
*13:02	1301 (1.13%)	165 (0.73%)	3.36E-09	5.54E-08	1.6	1.35-1.89	1.44%
*15:01	336 (0.29%)	69 (0.3%)	8.42E-01	9.26E-01	0.98	0.75-1.29	4.12%
*15:02	3306 (2.88%)	745 (3.29%)	4.08E-03	1.23E-02	0.89	0.82-0.96	4.22%
*15:11	311 (0.27%)	51 (0.23%)	2.04E-01	3.05E-01	1.23	0.91-1.68	0.67%
*15:18	327 (0.28%)	53 (0.23%)	1.68E-01	2.64E-01	1.24	0.93-1.69	0.63%
*15:25	575 (0.5%)	105 (0.46%)	3.81E-01	5.47E-01	1.1	0.89-1.37	0.76%
*27:04	2608 (2.27%)	562 (2.48%)	1.26E-01	2.19E-01	0.93	0.85-1.02	3.13%
*35:01	1356 (1.18%)	236 (1.04%)	4.18E-02	1.06E-01	1.16	1.01-1.34	2.78%
*35:03	79 (0.07%)	8 (0.04%)	6.15E-02	1.35E-01	1.98	0.96-4.76	0.12%

*37:01	311 (0.27%)	46 (0.2%)	5.47E-02	1.29E-01	1.36	0.99-1.9	0.37%
*38:02	3376 (2.94%)	811 (3.58%)	3.95E-06	3.26E-05	0.83	0.76-0.9	4.25%
*39:01	6 (0.01%)	2 (0.01%)	6.28E-01	7.98E-01	0.6	0.11-6.11	2.73%
*40:01	17757 (15.45%)	3851 (17.02%)	6.09E-07	6.70E-06	0.9	0.86-0.94	20.96%
*40:02	1157 (1.01%)	232 (1.03%)	1.00E+00	1.06E+00	1	0.87-1.16	2.19%
*40:06	1059 (0.92%)	178 (0.79%)	2.60E-02	7.14E-02	1.2	1.02-1.41	1.27%
*44:02	124 (0.11%)	10 (0.04%)	2.44E-03	8.96E-03	2.49	1.31-5.33	0.21%
*44:03	220 (0.19%)	19 (0.08%)	1.18E-04	6.49E-04	2.33	1.46-3.95	0.39%
*46:01	11219 (9.76%)	2430 (10.74%)	2.58E-04	1.22E-03	0.91	0.87-0.96	13.52%
*48:01	480 (0.42%)	77 (0.34%)	6.82E-02	1.41E-01	1.25	0.98-1.62	1.48%
*50:01	53 (0.05%)	9 (0.04%)	7.36E-01	8.37E-01	1.18	0.58-2.73	0.09%
*51:01	2549 (2.22%)	432 (1.91%)	7.38E-04	3.04E-03	1.19	1.07-1.33	4.35%
*51:02	633 (0.55%)	149 (0.66%)	8.31E-02	1.61E-01	0.85	0.71-1.03	1.24%
*52:01	801 (0.7%)	108 (0.48%)	4.64E-05	3.07E-04	1.5	1.22-1.85	0.97%
*54:01	2368 (2.06%)	513 (2.27%)	1.15E-01	2.11E-01	0.92	0.84-1.02	3.91%
*55:02	660 (0.57%)	126 (0.56%)	6.31E-01	7.71E-01	1.05	0.87-1.29	3.47%
*56:01	165 (0.14%)	28 (0.12%)	4.41E-01	6.07E-01	1.18	0.79-1.84	0.47%
*56:03	126 (0.11%)	28 (0.12%)	5.90E-01	7.79E-01	0.9	0.6-1.42	0.35%
*56:04	7 (0.01%)	1 (0%)	1.00E+00	1.06E+00	1.41	0.18-63.37	0.12%
*57:01	125 (0.11%)	22 (0.1%)	6.58E-01	7.76E-01	1.14	0.72-1.89	0.17%
*58:01	9712 (8.45%)	1584 (7%)	2.99E-17	9.85E-16	1.27	1.2-1.34	10.77%
*67:01	0 (0%)	0 (0%)	NA	NA	NA	NA	0.24%

[a]*P* value for Fisher's exact test.
[b]Q value for FDR-BH adjustment.

Table S7.6. Results of HLA-B subtypes significantly associated with HCV infection in the Taiwanese population. Abbreviations: OR, odds ratio; CI, confidence interval; N, allele number; NA, not available.

Genotype	*HCVab-*	*HCVab+*	*P-value*[a]	*Q-value*[b]	*OR*	*95% CI*	*Allele Frequency*
	N=113,974	*N=10,048*					
*07:02	179 (0.16%)	24 (0.24%)	7.15E-02	3.93E-01	0.67	0.43-1.07	0.35%
*13:01	5204 (4.57%)	477 (4.75%)	5.97E-01	9.86E-01	0.97	0.88-1.08	6.39%
*13:02	1198 (1.05%)	122 (1.21%)	1.72E-01	6.30E-01	0.88	0.73-1.07	1.44%
*15:01	339 (0.3%)	34 (0.34%)	5.08E-01	9.86E-01	0.89	0.62-1.31	4.12%
*15:02	3377 (2.96%)	269 (2.68%)	6.34E-02	4.18E-01	1.13	0.99-1.29	4.22%
*15:11	298 (0.26%)	22 (0.22%)	4.74E-01	9.78E-01	1.21	0.79-1.97	0.67%
*15:18	331 (0.29%)	18 (0.18%)	3.94E-02	4.33E-01	1.65	1.03-2.82	0.63%
*15:25	575 (0.5%)	53 (0.53%)	8.27E-01	1.14E+00	0.97	0.73-1.31	0.76%
*27:04	2704 (2.37%)	218 (2.17%)	1.39E-01	5.74E-01	1.11	0.97-1.29	3.13%
*35:01	1369 (1.2%)	95 (0.95%)	1.41E-02	2.33E-01	1.29	1.05-1.61	2.78%
*35:03	72 (0.06%)	5 (0.05%)	8.34E-01	1.10E+00	1.29	0.53-4.09	0.12%
*37:01	302 (0.26%)	23 (0.23%)	5.43E-01	9.43E-01	1.18	0.77-1.88	0.37%
*38:02	3420 (3%)	326 (3.24%)	2.59E-01	7.76E-01	0.94	0.83-1.05	4.25%

*39:01	6 (0.01%)	0 (0%)	NA	NA	NA	NA	2.73%
*40:01	17939 (15.74%)	1586 (15.78%)	6.16E-01	9.69E-01	1.02	0.96-1.08	20.96%
*40:02	1144 (1%)	104 (1.04%)	8.76E-01	1.11E+00	0.98	0.8-1.22	2.19%
*40:06	1048 (0.92%)	80 (0.8%)	1.89E-01	6.25E-01	1.17	0.93-1.5	1.27%
*44:02	110 (0.1%)	11 (0.11%)	7.39E-01	1.11E+00	0.89	0.48-1.84	0.21%
*44:03	190 (0.17%)	20 (0.2%)	4.51E-01	9.92E-01	0.85	0.53-1.42	0.39%
*46:01	11351 (9.96%)	957 (9.52%)	5.45E-02	4.49E-01	1.07	1-1.15	13.52%
*48:01	461 (0.4%)	40 (0.4%)	9.35E-01	1.10E+00	1.03	0.74-1.46	1.48%
*50:01	51 (0.04%)	4 (0.04%)	1.00E+00	1.14E+00	1.14	0.42-4.35	0.09%
*51:01	2443 (2.14%)	223 (2.22%)	7.74E-01	1.11E+00	0.98	0.85-1.13	4.35%
*51:02	645 (0.57%)	64 (0.64%)	4.10E-01	1.04E+00	0.9	0.69-1.18	1.24%
*52:01	746 (0.65%)	80 (0.8%)	1.26E-01	5.93E-01	0.83	0.66-1.06	0.97%
*54:01	2351 (2.06%)	223 (2.22%)	4.01E-01	1.10E+00	0.94	0.82-1.09	3.91%
*55:02	653 (0.57%)	63 (0.63%)	5.38E-01	9.87E-01	0.93	0.71-1.22	3.47%
*56:01	162 (0.14%)	15 (0.15%)	8.91E-01	1.09E+00	0.97	0.57-1.77	0.47%
*56:03	126 (0.11%)	14 (0.14%)	4.39E-01	1.03E+00	0.8	0.46-1.52	0.35%
*56:04	8 (0.01%)	0 (0%)	NA	NA	NA	NA	0.12%
*57:01	117 (0.1%)	10 (0.1%)	1.00E+00	1.14E+00	1.05	0.55-2.24	0.17%
*58:01	9277 (8.14%)	920 (9.16%)	1.45E-03	4.78E-02	0.89	0.82-0.96	10.77%
*67:01	0 (0%)	0 (0%)	NA	NA	NA	NA	0.24%

[a]*P* value for Fisher's exact test.
[b]Q value for FDR-BH adjustment.

Table S7.7. Results of HLA-C subtypes significantly associated with HCC in the Taiwanese population. Abbreviations: OR, odds ratio; CI, confidence interval; N, allele number; NA, not available.

Genotype	*Control*	*Case*	*P-value*[a]	*Q-value*[b]	*OR*	*95% CI*	*Allele Frequency*
	N=241,306	*N=5,858*					
*01:02	38654 (16.02%)	1017 (17.36%)	2.90E-02	5.50E-01	0.92	0.86-0.99	20.08%
*02:02	3 (0%)	0 (0%)	NA	NA	NA	NA	0.11%
*03:02	23448 (9.72%)	552 (9.42%)	2.34E-01	6.34E-01	1.06	0.97-1.16	10.80%
*03:03	8447 (3.5%)	179 (3.06%)	3.72E-02	2.36E-01	1.17	1.01-1.37	4.91%
*03:04	24239 (10.04%)	607 (10.36%)	7.40E-01	8.78E-01	0.99	0.9-1.08	12.70%
*04:01	6799 (2.82%)	175 (2.99%)	5.78E-01	1.10E+00	0.96	0.82-1.12	3.80%
*04:03	3348 (1.39%)	77 (1.31%)	5.74E-01	1.21E+00	1.08	0.86-1.37	1.73%
*04:82	906 (0.38%)	24 (0.41%)	6.69E-01	1.06E+00	0.93	0.62-1.47	0.64%
*06:02	4405 (1.83%)	110 (1.88%)	8.83E-01	9.87E-01	0.99	0.82-1.21	2.06%
*07:02	39824 (16.5%)	1021 (17.43%)	1.97E-01	6.25E-01	0.95	0.89-1.03	21.20%
*07:04	813 (0.34%)	18 (0.31%)	7.34E-01	9.30E-01	1.12	0.7-1.9	0.66%
*07:06	364 (0.15%)	10 (0.17%)	7.34E-01	9.96E-01	0.9	0.48-1.89	0.19%
*08:01	12202 (5.06%)	295 (5.04%)	7.18E-01	1.05E+00	1.02	0.91-1.16	7.55%
*12:02	8716 (3.61%)	193 (3.29%)	1.28E-01	4.88E-01	1.12	0.97-1.3	4.15%
*12:03	1462 (0.61%)	35 (0.6%)	9.33E-01	9.84E-01	1.03	0.74-1.49	0.92%

*14:02	7233 (3%)	151 (2.58%)	3.67E-02	3.48E-01	1.19	1.01-1.41	3.51%
*14:03	193 (0.08%)	1 (0.02%)	9.77E-02	4.64E-01	4.77	0.85-189.5	0.09%
*15:02	7470 (3.1%)	197 (3.36%)	3.62E-01	8.60E-01	0.93	0.81-1.09	4.06%
*16:02	50 (0.02%)	0 (0%)	NA	NA	NA	NA	0.13%

[a]*P* value for Fisher's exact test.
[b]Q value for FDR-BH adjustment.

Table S7.8. Results of HLA-C subtypes significantly associated with HBV infection in the Taiwanese population. Abbreviations: OR, odds ratio; CI, confidence interval; N, allele number; NA, not available.

Genotype	*HBSag-*	*HBSag+*	*P-value*[a]	*Q-value*[b]	*OR*	*95% CI*	*Allele Frequency*
	N=114,898	*N=22,632*					
*01:02	18549 (16.14%)	3923 (17.33%)	4.40E-05	1.19E-04	0.92	0.89-0.96	20.08%
*02:02	1 (0%)	0 (0%)	NA	NA	NA	NA	0.11%
*03:02	11302 (9.84%)	1853 (8.19%)	1.52E-16	2.89E-15	1.24	1.18-1.31	10.80%
*03:03	3957 (3.44%)	777 (3.43%)	8.10E-01	1.03E+00	1.01	0.93-1.09	4.91%
*03:04	11453 (9.97%)	2440 (10.78%)	6.45E-04	1.36E-03	0.92	0.88-0.97	12.70%
*04:01	3269 (2.85%)	549 (2.43%)	1.95E-04	4.63E-04	1.19	1.08-1.3	3.80%
*04:03	1605 (1.4%)	320 (1.41%)	9.26E-01	1.03E+00	0.99	0.88-1.13	1.73%
*04:82	487 (0.42%)	79 (0.35%)	1.00E-01	1.90E-01	1.22	0.96-1.57	0.64%
*06:02	2126 (1.85%)	292 (1.29%)	5.32E-10	5.05E-09	1.45	1.29-1.65	2.06%
*07:02	18795 (16.36%)	4011 (17.72%)	2.53E-06	1.60E-05	0.91	0.88-0.95	21.20%
*07:04	414 (0.36%)	77 (0.34%)	6.70E-01	9.09E-01	1.07	0.83-1.38	0.66%
*07:06	162 (0.14%)	23 (0.1%)	1.38E-01	2.39E-01	1.4	0.9-2.27	0.19%
*08:01	5764 (5.02%)	1140 (5.04%)	9.47E-01	9.99E-01	1	0.94-1.07	7.55%
*12:02	4289 (3.73%)	834 (3.69%)	6.03E-01	8.81E-01	1.02	0.95-1.1	4.15%
*12:03	701 (0.61%)	136 (0.6%)	8.52E-01	1.01E+00	1.02	0.85-1.24	0.92%
*14:02	3496 (3.04%)	569 (2.51%)	5.59E-06	2.66E-05	1.23	1.12-1.35	3.51%
*14:03	89 (0.08%)	2 (0.01%)	2.57E-05	8.14E-05	8.83	2.37-73.97	0.09%
*15:02	3515 (3.06%)	825 (3.65%)	1.30E-05	4.96E-05	0.84	0.78-0.91	4.06%
*16:02	28 (0.02%)	2 (0.01%)	2.16E-01	3.42E-01	2.78	0.7-24.06	0.13%

[a]*P* value for Fisher's exact test.
[b]Q value for FDR-BH adjustment.

Table S7.9. Results of HLA-C subtypes significantly associated with HCV infection in the Taiwanese population. Abbreviations: OR, odds ratio; CI, confidence interval; N, allele number; NA, not available.

Genotype	*HCVab-*	*HCVab+*	*P-value*[a]	*Q-value*[b]	*OR*	*95% CI*	*Allele Frequency*
	N=113,974	*N=10,048*					
*01:02	18614 (16.33%)	1605 (15.97%)	2.66E-01	7.21E-01	1.03	0.98-1.09	20.08%
*02:02	1 (0%)	0 (0%)	NA	NA	NA	NA	0.11%
*03:02	10796 (9.47%)	1076 (10.71%)	9.32E-05	1.77E-03	0.87	0.82-0.93	10.80%

*03:03	3905 (3.43%)	356 (3.54%)	5.86E-01	1.11E+00	0.97	0.87-1.09	4.91%
*03:04	11423 (10.02%)	1064 (10.59%)	8.89E-02	4.22E-01	0.94	0.88-1.01	12.70%
*04:01	3147 (2.76%)	271 (2.7%)	6.79E-01	1.07E+00	1.03	0.91-1.17	3.80%
*04:03	1626 (1.43%)	135 (1.34%)	5.09E-01	1.21E+00	1.07	0.89-1.28	1.73%
*04:82	486 (0.43%)	44 (0.44%)	8.73E-01	1.19E+00	0.98	0.72-1.36	0.64%
*06:02	1999 (1.75%)	185 (1.84%)	5.53E-01	1.17E+00	0.96	0.82-1.12	2.06%
*07:02	18844 (16.53%)	1678 (16.7%)	7.85E-01	1.15E+00	0.99	0.94-1.05	21.20%
*07:04	427 (0.37%)	22 (0.22%)	9.32E-03	5.90E-02	1.72	1.12-2.78	0.66%
*07:06	149 (0.13%)	13 (0.13%)	1.00E+00	1.27E+00	1.01	0.58-1.95	0.19%
*08:01	5797 (5.09%)	453 (4.51%)	7.91E-03	7.51E-02	1.14	1.03-1.26	7.55%
*12:02	4330 (3.8%)	353 (3.51%)	1.32E-01	4.17E-01	1.09	0.98-1.22	4.15%
*12:03	714 (0.63%)	58 (0.58%)	5.97E-01	1.03E+00	1.09	0.83-1.45	0.92%
*14:02	3327 (2.92%)	294 (2.93%)	1.00E+00	1.27E+00	1	0.89-1.13	3.51%
*14:03	72 (0.06%)	6 (0.06%)	1.00E+00	1.27E+00	1.06	0.46-2.99	0.09%
*15:02	3664 (3.21%)	293 (2.92%)	9.05E-02	3.44E-01	1.11	0.98-1.26	4.06%
*16:02	27 (0.02%)	2 (0.02%)	1.00E+00	1.27E+00	1.19	0.3-10.37	0.13%

[a]*P* value for Fisher's exact test.
[b]Q value for FDR-BH adjustment.

Table S7.10. Results of HLA-DPA1 subtypes significantly associated with HCC in the Taiwanese population. Abbreviations: OR, odds ratio; CI, confidence interval; N, allele number.

Genotype	*Control*	*Case*	*P-value*[a]	*Q-value*[b]	*OR*	*95% CI*	*Allele Frequency*
	N=241,306	*N=5,858*					
*01:03	66546 (27.58%)	1484 (25.33%)	9.95E-05	1.99E-04	1.13	1.06-1.2	27.75%
*02:01	16511 (6.84%)	387 (6.61%)	4.79E-01	6.39E-01	1.04	0.94-1.16	7.56%
*02:02	147230 (61.01%)	3725 (63.59%)	8.18E-05	3.27E-04	0.9	0.85-0.95	61.89%
*04:01	6263 (2.6%)	154 (2.63%)	8.68E-01	8.68E-01	0.99	0.84-1.17	2.81%

[a]*P* value for Fisher's exact test.
[b]Q value for FDR-BH adjustment.

Table S7.11. Results of HLA-DPA1 subtypes significantly associated with HBV infection in the Taiwanese population. Abbreviations: OR, odds ratio; CI, confidence interval; N, allele number.

Genotype	*HBSag-*	*HBSag+*	*P-value*[a]	*Q-value*[b]	*OR*	*95% CI*	*Allele Frequency*
	N=114,898	*N=22,632*					
*01:03	32061 (27.9%)	4897 (21.64%)	1.30E-86	2.60E-86	1.4	1.35-1.45	27.75%
*02:01	7933 (6.9%)	1314 (5.81%)	1.31E-09	1.75E-09	1.2	1.13-1.28	7.56%
*02:02	69750 (60.71%)	15266 (67.45%)	2.24E-88	8.94E-88	0.73	0.71-0.76	61.89%
*04:01	2888 (2.51%)	671 (2.96%)	1.06E-04	1.06E-04	0.84	0.77-0.92	2.81%

[a]*P* value for Fisher's exact test.
[b]Q value for FDR-BH adjustment.

Table S7.12. Results of HLA-DPA1 subtypes significantly associated with HCV infection in the Taiwanese population. Abbreviations: OR, odds ratio; CI, confidence interval; N, allele number.

Genotype	*HCVab-*	*HCVab+*	*P-value*[a]	*Q-value*[b]	*OR*	*95% CI*	*Allele Frequency*
	N=113,974	*N=10,048*					
*01:03	30443 (26.71%)	2806 (27.93%)	1.29E-02	5.18E-02	0.94	0.9-0.99	27.75%
*02:01	7639 (6.7%)	683 (6.8%)	7.55E-01	7.55E-01	0.99	0.91-1.07	7.56%
*02:02	70644 (61.98%)	6143 (61.14%)	4.52E-02	9.04E-02	1.04	1-1.09	61.89%
*04:01	2976 (2.61%)	240 (2.39%)	1.80E-01	2.40E-01	1.1	0.96-1.26	2.81%

[a]*P* value for Fisher's exact test.
[b]Q value for FDR-BH adjustment.

Table S7.13. Results of HLA-DPB1 subtypes significantly associated with HCC in the Taiwanese population. Abbreviations: OR, odds ratio; CI, confidence interval; N, allele number; NA, not available.

Genotype	*Control*	*Case*	*P-value*[a]	*Q-value*[b]	*OR*	*95% CI*	*Allele Frequency*
	N=241,306	*N=5,858*					
*01:01	55 (0.02%)	1 (0.02%)	1.00E+00	1.08E+00	1.37	0.24-55.13	0.21%
*02:01	25815 (10.70%)	568 (9.70%)	1.35E-03	9.45E-03	1.16	1.06-1.27	15.29%
*02:02	10848 (4.50%)	242 (4.13%)	8.00E-02	2.80E-01	1.12	0.99-1.29	7.33%
*03:01	7889 (3.27%)	177 (3.02%)	1.71E-01	3.42E-01	1.12	0.96-1.31	4.74%
*04:01	14239 (5.90%)	346 (5.91%)	6.54E-01	9.15E-01	1.03	0.92-1.15	7.77%
*04:02	2640 (1.09%)	47 (0.80%)	1.93E-02	9.03E-02	1.41	1.05-1.92	1.76%
*05:01	87978 (36.46%)	2347 (40.06%)	3.72E-06	5.21E-05	0.87	0.82-0.92	45.15%
*09:01	2334 (0.97%)	45 (0.77%)	9.34E-02	2.61E-01	1.30	0.96-1.79	1.38%
*104:01	194 (0.08%)	4 (0.07%)	1.00E+00	1.08E+00	1.21	0.46-4.48	0.32%
*13:01	11341 (4.70%)	309 (5.27%)	1.13E-01	2.63E-01	0.91	0.81-1.03	6.66%
*135:01	2 (0.0008%)	0 (0%)	NA	NA	NA	NA	2.51%
*14:01	3576 (1.48%)	83 (1.42%)	5.51E-01	8.57E-01	1.08	0.86-1.36	2.56%
*17:01	1914 (0.79%)	39 (0.67%)	2.37E-01	4.14E-01	1.23	0.89-1.73	0.90%
*19:01	2669 (1.11%)	69 (1.18%)	7.54E-01	9.60E-01	0.96	0.76-1.24	1.33%
*21:01	2734 (1.13%)	65 (1.11%)	7.57E-01	8.83E-01	1.05	0.82-1.37	1.79%

[a]*P* value for Fisher's exact test.
[b]Q value for FDR-BH adjustment.

Table S7.14. Results of HLA-DPB1 subtypes significantly associated with HBV infection in the Taiwanese population. Abbreviations: OR, odds ratio; CI, confidence interval; N, allele number; NA, not available.

Genotype	*HBSag-*	*HBSag+*	*P-value*[a]	*Q-value*[b]	*OR*	*95% CI*	*Allele Frequency*
	N=114,898	*N=22,632*					
*01:01	22 (0.02%)	3 (0.01%)	7.89E-01	7.89E-01	1.50	0.45-7.81	0.21%
*02:01	12517 (10.89%)	1983 (8.76%)	5.25E-31	2.45E-30	1.34	1.27-1.41	15.29%
*02:02	5312 (4.62%)	696 (3.08%)	9.26E-33	6.48E-32	1.59	1.47-1.73	7.33%

*03:01	3903 (3.40%)	581 (2.57%)	7.03E-14	1.41E-13	1.39	1.27-1.52	4.74%
*04:01	6770 (5.89%)	980 (4.33%)	1.98E-27	6.94E-27	1.45	1.35-1.55	7.77%
*04:02	1284 (1.12%)	185 (0.82%)	4.26E-06	6.63E-06	1.42	1.22-1.67	1.76%
*05:01	41503 (36.12%)	10002 (44.19%)	1.11E-103	1.56E-102	0.69	0.67-0.72	45.15%
*09:01	1149 (1.00%)	113 (0.50%)	3.00E-16	8.39E-16	2.09	1.72-2.56	1.38%
*104:01	94 (0.08%)	2 (0.01%)	7.32E-06	1.03E-05	9.59	2.58-80.39	0.32%
*13:01	5191 (4.52%)	1347 (5.95%)	1.55E-15	3.61E-15	0.77	0.72-0.82	6.66%
*135:01	2 (0.002%)	0 (0%)	NA	NA	NA	NA	2.51%
*14:01	1759 (1.53%)	300 (1.33%)	3.61E-03	4.21E-03	1.20	1.06-1.36	2.56%
*17:01	909 (0.79%)	98 (0.43%)	8.31E-11	1.45E-10	1.90	1.54-2.37	0.90%
*19:01	1241 (1.08%)	332 (1.47%)	1.64E-05	2.09E-05	0.76	0.67-0.86	1.33%
*21:01	1310 (1.14%)	298 (1.32%)	8.74E-02	9.41E-02	0.89	0.79-1.02	1.79%

[a]*P* value for Fisher's exact test.
[b]Q value for FDR-BH adjustment.

Table S7.15. Results of HLA-DPB1 subtypes significantly associated with HCV infection in the Taiwanese population. Abbreviations: OR, odds ratio; CI, confidence interval; N, allele number; NA, not available.

Genotype	*HCVab-*	*HCVab+*	*P-value*[a]	*Q-value*[b]	*OR*	*95% CI*	*Allele Frequency*
	N=113,974	*N=10,048*					
*01:01	19 (0.02%)	3 (0.03%)	4.20E-01	4.90E-01	0.56	0.17-2.98	0.21%
*02:01	11991 (10.52%)	1103 (10.98%)	2.85E-01	4.43E-01	0.96	0.90-1.03	15.29%
*02:02	4911 (4.31%)	483 (4.81%)	3.57E-02	9.99E-02	0.90	0.82-0.99	7.33%
*03:01	3702 (3.25%)	336 (3.34%)	7.47E-01	7.47E-01	0.98	0.88-1.10	4.74%
*04:01	6334 (5.56%)	628 (6.25%)	8.86E-03	1.24E-01	0.89	0.82-0.97	7.77%
*04:02	1173 (1.03%)	114 (1.13%)	3.84E-01	4.88E-01	0.92	0.75-1.12	1.76%
*05:01	42827 (37.58%)	3704 (36.86%)	1.10E-02	7.73E-02	1.06	1.01-1.12	45.15%
*09:01	1022 (0.90%)	107 (1.06%)	1.13E-01	2.64E-01	0.85	0.69-1.05	1.38%
*104:01	75 (0.07%)	14 (0.14%)	1.77E-02	8.26E-02	0.48	0.27-0.91	0.32%
*13:01	5486 (4.81%)	438 (4.36%)	2.12E-02	7.41E-02	1.13	1.02-1.25	6.66%
*135:01	0 (0%)	0 (0%)	NA	NA	NA	NA	2.51%
*14:01	1719 (1.51%)	140 (1.39%)	3.25E-01	4.55E-01	1.10	0.92-1.31	2.56%
*17:01	810 (0.71%)	86 (0.86%)	1.25E-01	2.51E-01	0.84	0.67-1.06	0.90%
*19:01	1319 (1.16%)	105 (1.04%)	2.84E-01	4.96E-01	1.12	0.92-1.38	1.33%
*21:01	1342 (1.18%)	115 (1.14%)	7.36E-01	7.92E-01	1.04	0.86-1.27	1.79%

[a]*P* value for Fisher's exact test.
[b]Q value for FDR-BH adjustment.

Table S7.16. Results of HLA-DQA1 subtypes significantly associated with HCC in the Taiwanese population. Abbreviations: OR, odds ratio; CI, confidence interval; N, allele number; NA, not available.

Genotype	*Control*	*Case*	*P-value*[a]	*Q-value*[b]	*OR*	*95% CI*	*Allele Frequency*
	N=241,306	*N=5,858*					

*01:01	3455 (1.43%)	94 (1.6%)	2.92E-01	5.47E-01	0.9	0.73-1.11	1.97%
*01:02	36028 (14.93%)	773 (13.2%)	6.70E-05	3.35E-04	1.17	1.08-1.26	16.45%
*01:03	20562 (8.52%)	510 (8.71%)	7.21E-01	8.32E-01	0.98	0.9-1.08	9.23%
*01:04	15020 (6.22%)	424 (7.24%)	2.82E-03	8.45E-03	0.85	0.77-0.95	7.05%
*01:05	2158 (0.89%)	56 (0.96%)	6.24E-01	7.80E-01	0.94	0.72-1.25	1.08%
*02:01	4183 (1.73%)	101 (1.72%)	9.60E-01	1.03E+00	1.01	0.83-1.25	2.15%
*03:01	13708 (5.68%)	263 (4.49%)	3.55E-05	2.66E-04	1.29	1.14-1.47	7.35%
*03:02	33268 (13.79%)	785 (13.4%)	3.04E-01	5.07E-01	1.04	0.96-1.13	15.29%
*03:03	12600 (5.22%)	271 (4.63%)	3.18E-02	7.95E-02	1.14	1.01-1.3	7.94%
*04:01	2438 (1.01%)	35 (0.6%)	8.81E-04	3.30E-03	1.71	1.22-2.46	1.19%
*05:01	17825 (7.39%)	449 (7.66%)	5.10E-01	6.95E-01	0.97	0.88-1.07	8.23%
*05:03	32 (0.01%)	0 (0%)	NA	NA	NA	NA	0.63%
*05:05	23587 (9.77%)	597 (10.19%)	3.70E-01	5.55E-01	0.96	0.88-1.05	11.44%
*05:08	999 (0.41%)	31 (0.53%)	1.83E-01	3.92E-01	0.79	0.55-1.16	0.80%
*06:01	20085 (8.32%)	643 (10.98%)	7.72E-12	1.16E-10	0.74	0.68-0.8	9.20%

[a]*P* value for Fisher's exact test.
[b]Q value for FDR-BH adjustment.

Table S7.17. Results of HLA-DQA1 haplotypes significantly associated with HBV infection in the Taiwanese population. Abbreviations: OR, odds ratio; CI, confidence interval; N, allele number.

Genotype	*HBSag-*	*HBSag+*	*P-value*[a]	*Q-value*[b]	*OR*	*95% CI*	*Allele Frequency*
	N=114,898	*N=22,632*					
*01:01	1682 (1.46%)	280 (1.24%)	1.73E-03	2.60E-03	1.22	1.08-1.39	1.97%
*01:02	17433 (15.17%)	2835 (12.53%)	1.52E-34	7.58E-34	1.3	1.25-1.36	16.45%
*01:03	9821 (8.55%)	1971 (8.71%)	6.50E-01	7.50E-01	1.01	0.96-1.07	9.23%
*01:04	7130 (6.21%)	1764 (7.79%)	2.54E-14	6.36E-14	0.81	0.76-0.85	7.05%
*01:05	1043 (0.91%)	137 (0.61%)	5.30E-07	9.94E-07	1.55	1.3-1.87	1.08%
*02:01	1995 (1.74%)	235 (1.04%)	1.71E-17	5.14E-17	1.74	1.52-2	2.15%
*03:01	6563 (5.71%)	762 (3.37%)	1.48E-58	2.22E-57	1.8	1.67-1.95	7.35%
*03:02	15610 (13.59%)	3711 (16.4%)	7.41E-21	2.78E-20	0.83	0.79-0.86	15.29%
*03:03	6147 (5.35%)	1259 (5.56%)	7.49E-01	8.02E-01	0.99	0.93-1.05	7.94%
*04:01	1130 (0.98%)	190 (0.84%)	1.62E-02	2.03E-02	1.21	1.04-1.42	1.19%
*05:01	8407 (7.32%)	1514 (6.69%)	6.85E-06	1.14E-05	1.14	1.08-1.21	8.23%
*05:03	13 (0.01%)	2 (0.01%)	1.00E+00	1.00E+00	1.32	0.3-12.04	0.63%
*05:05	10891 (9.48%)	2503 (11.06%)	3.43E-09	7.36E-09	0.87	0.83-0.91	11.44%
*05:08	454 (0.4%)	127 (0.56%)	1.83E-03	2.50E-03	0.72	0.59-0.89	0.80%
*06:01	9493 (8.26%)	2554 (11.28%)	6.91E-39	5.18E-38	0.73	0.69-0.76	9.20%

[a]*P* value for Fisher's exact test.
[b]Q value for FDR-BH adjustment.

Table S7.18. Results of HLA-DQA1 subtypes significantly associated with HCV infection in the Taiwanese population. Abbreviations: OR, odds ratio; CI, confidence interval; N, allele number.

Genotype	*HCVab-*	*HCVab+*	*P-value*[a]	*Q-value*[b]	*OR*	*95% CI*	*Allele Frequency*
	N=113,974	*N=10,048*					
*01:01	1603 (1.41%)	148 (1.47%)	5.36E-01	6.70E-01	0.95	0.8-1.13	1.97%
*01:02	16742 (14.69%)	1529 (15.22%)	1.04E-01	3.11E-01	0.95	0.9-1.01	16.45%
*01:03	9807 (8.6%)	807 (8.03%)	6.56E-02	2.46E-01	1.07	1-1.16	9.23%
*01:04	7354 (6.45%)	664 (6.61%)	4.68E-01	6.39E-01	0.97	0.89-1.05	7.05%
*01:05	975 (0.86%)	100 (1%)	1.43E-01	3.06E-01	0.85	0.69-1.06	1.08%
*02:01	1817 (1.59%)	191 (1.9%)	1.83E-02	1.37E-01	0.83	0.71-0.97	2.15%
*03:01	6067 (5.32%)	552 (5.49%)	4.01E-01	6.69E-01	0.96	0.88-1.05	7.35%
*03:02	16056 (14.09%)	1357 (13.51%)	1.48E-01	2.78E-01	1.05	0.98-1.11	15.29%
*03:03	6141 (5.39%)	527 (5.24%)	6.26E-01	7.22E-01	1.02	0.93-1.12	7.94%
*04:01	1098 (0.96%)	88 (0.88%)	4.53E-01	6.79E-01	1.1	0.88-1.38	1.19%
*05:01	8138 (7.14%)	800 (7.96%)	1.54E-03	2.32E-02	0.88	0.82-0.95	8.23%
*05:03	13 (0.01%)	1 (0.01%)	1.00E+00	1.00E+00	1.14	0.17-48.49	0.63%
*05:05	11166 (9.8%)	931 (9.27%)	1.14E-01	2.86E-01	1.06	0.99-1.14	11.44%
*05:08	477 (0.42%)	44 (0.44%)	7.47E-01	8.00E-01	0.95	0.7-1.33	0.80%
*06:01	10086 (8.85%)	821 (8.17%)	2.85E-02	1.43E-01	1.09	1.01-1.17	9.20%

[a]*P* value for Fisher's exact test.
[b]Q value for FDR-BH adjustment.

Table S7.19. Results of HLA-DQB1 subtypes significantly associated with HCC in the Taiwanese population. Abbreviations: OR, odds ratio; CI, confidence interval; N, allele number.

Genotype	*Control*	*Case*	*P-value*[a]	*Q-value*[b]	*OR*	*95% CI*	*Allele Frequency*
	N=241,306	*N=5,858*					
*02:01	18903 (7.83%)	477 (8.14%)	4.02E-01	5.48E-01	0.96	0.87-1.06	8.22%
*02:02	4325 (1.79%)	108 (1.84%)	7.65E-01	8.83E-01	0.97	0.8-1.19	1.93%
*03:01	50703 (21.01%)	1445 (24.67%)	2.89E-11	4.34E-10	0.81	0.76-0.86	22.38%
*03:02	17987 (7.45%)	331 (5.65%)	5.91E-08	4.43E-07	1.35	1.21-1.51	7.93%
*03:03	35412 (14.68%)	818 (13.96%)	1.14E-01	2.45E-01	1.06	0.99-1.15	15.51%
*04:01	15231 (6.31%)	361 (6.16%)	6.43E-01	8.04E-01	1.03	0.92-1.15	7.06%
*04:02	2357 (0.98%)	34 (0.58%)	1.44E-03	4.33E-03	1.69	1.21-2.45	1.21%
*05:01	5175 (2.14%)	138 (2.36%)	2.74E-01	5.13E-01	0.91	0.77-1.09	2.59%
*05:02	22449 (9.3%)	588 (10.04%)	6.13E-02	1.53E-01	0.92	0.84-1.01	10.02%
*05:03	9312 (3.86%)	227 (3.88%)	9.73E-01	9.73E-01	1	0.87-1.15	4.51%
*06:01	27786 (11.51%)	669 (11.42%)	8.03E-01	8.60E-01	1.01	0.93-1.1	12.00%
*06:02	8287 (3.43%)	148 (2.53%)	8.15E-05	4.08E-04	1.38	1.17-1.63	3.89%
*06:03	523 (0.22%)	9 (0.15%)	3.90E-01	5.85E-01	1.41	0.74-3.11	0.37%
*06:04	150 (0.06%)	1 (0.02%)	2.75E-01	4.59E-01	3.65	0.64-145.05	0.14%
*06:09	4624 (1.92%)	74 (1.26%)	1.53E-04	5.75E-04	1.53	1.21-1.96	2.04%

[a]*P* value for Fisher's exact test.
[b]Q value for FDR-BH adjustment.

Table S7.20. Results of HLA-DQB1 subtypes significantly associated with HBV infection in the Taiwanese population. Abbreviations: OR, odds ratio; CI, confidence interval; N, allele number.

Genotype	*HBSag-*	*HBSag+*	*P-value*[a]	*Q-value*[b]	*OR*	*95% CI*	*Allele Frequency*
	N=114,898	*N=22,632*					
*02:01	8904 (7.75%)	1587 (7.01%)	1.05E-05	1.58E-05	1.13	1.07-1.2	8.22%
*02:02	2061 (1.79%)	246 (1.09%)	2.27E-16	5.67E-16	1.69	1.48-1.93	1.93%
*03:01	23529 (20.48%)	5766 (25.48%)	4.45E-55	2.22E-54	0.76	0.74-0.79	22.38%
*03:02	8702 (7.57%)	956 (4.22%)	4.48E-86	6.72E-85	1.89	1.77-2.03	7.93%
*03:03	16646 (14.49%)	3905 (17.25%)	2.05E-22	7.67E-22	0.82	0.79-0.86	15.51%
*04:01	7489 (6.52%)	1468 (6.49%)	4.99E-01	4.99E-01	1.02	0.96-1.08	7.06%
*04:02	1085 (0.94%)	182 (0.8%)	2.79E-02	3.22E-02	1.19	1.02-1.4	1.21%
*05:01	2508 (2.18%)	376 (1.66%)	6.97E-08	1.16E-07	1.34	1.2-1.5	2.59%
*05:02	10831 (9.43%)	2454 (10.84%)	4.02E-09	7.53E-09	0.87	0.83-0.91	10.02%
*05:03	4417 (3.84%)	979 (4.33%)	2.98E-03	3.73E-03	0.9	0.84-0.96	4.51%
*06:01	13257 (11.54%)	2576 (11.38%)	1.71E-01	1.84E-01	1.03	0.99-1.08	12.00%
*06:02	3967 (3.45%)	537 (2.37%)	1.88E-19	5.65E-19	1.49	1.36-1.64	3.89%
*06:03	253 (0.22%)	11 (0.05%)	4.64E-10	9.95E-10	4.6	2.53-9.34	0.37%
*06:04	70 (0.06%)	3 (0.01%)	2.33E-03	3.18E-03	4.66	1.53-23.16	0.14%
*06:09	2315 (2.01%)	136 (0.6%)	1.73E-63	1.30E-62	3.45	2.9-4.14	2.04%

[a]*P* value for Fisher's exact test.
[b]Q value for FDR-BH adjustment.

Table S7.21. Results of HLA-DQB1 subtypes significantly associated with HCV infection in the Taiwanese population. Abbreviations: OR, odds ratio; CI, confidence interval; N, allele number.

Genotype	*HCVab-*	*HCVab+*	*P-value*[a]	*Q-value*[b]	*OR*	*95% CI*	*Allele Frequency*
	N=113,974	*N=10,048*					
*02:01	8585 (7.53%)	854 (8.5%)	2.81E-04	4.22E-03	0.87	0.81-0.94	8.22%
*02:02	1879 (1.65%)	193 (1.92%)	3.77E-02	1.41E-01	0.85	0.73-0.99	1.93%
*03:01	24477 (21.48%)	1993 (19.83%)	2.83E-04	2.12E-03	1.1	1.04-1.16	22.38%
*03:02	7971 (6.99%)	740 (7.36%)	1.25E-01	3.12E-01	0.94	0.87-1.02	7.93%
*03:03	17055 (14.96%)	1440 (14.33%)	1.36E-01	2.56E-01	1.05	0.99-1.11	15.51%
*04:01	7424 (6.51%)	690 (6.87%)	1.28E-01	2.74E-01	0.94	0.87-1.02	7.06%
*04:02	1052 (0.92%)	86 (0.86%)	5.84E-01	6.74E-01	1.07	0.86-1.35	1.21%
*05:01	2375 (2.08%)	219 (2.18%)	4.66E-01	6.35E-01	0.95	0.83-1.1	2.59%
*05:02	10916 (9.58%)	958 (9.53%)	9.72E-01	9.72E-01	1	0.93-1.07	10.02%
*05:03	4475 (3.93%)	398 (3.96%)	7.67E-01	8.22E-01	0.99	0.89-1.1	4.51%
*06:01	13165 (11.55%)	1095 (10.9%)	7.58E-02	2.28E-01	1.06	0.99-1.13	12.00%
*06:02	3753 (3.29%)	342 (3.4%)	4.83E-01	6.04E-01	0.96	0.86-1.08	3.89%
*06:03	229 (0.2%)	15 (0.15%)	3.45E-01	5.17E-01	1.34	0.79-2.43	0.37%

*06:04	56 (0.05%)	8 (0.08%)	1.72E-01	2.87E-01	0.61	0.29-1.49	0.14%
*06:09	2036 (1.79%)	213 (2.12%)	1.52E-02	7.61E-02	0.83	0.72-0.97	2.04%

[a]*P* value for Fisher's exact test.
[b]Q value for FDR-BH adjustment.

Table S7.22. Results of HLA-DRB1 subtypes significantly associated with HCC in the Taiwanese population. Abbreviations: OR, odds ratio; CI, confidence interval; N, allele number.

Genotype	*Control*	*Case*	*P-value[a]*	*Q-value[b]*	*OR*	*95% CI*	*Allele Frequency*
	N=241,306	*N=5,858*					
*01:01	863 (0.36%)	19 (0.32%)	5.90E-01	7.87E-01	1.18	0.75-1.98	0.45%
*03:01	16548 (6.86%)	424 (7.24%)	7.38E-01	8.68E-01	1.02	0.92-1.13	8.23%
*04:01	225 (0.09%)	3 (0.05%)	3.93E-01	8.73E-01	1.96	0.66-9.56	0.24%
*04:03	76 (0.03%)	2 (0.03%)	1.00E+00	1.00E+00	0.99	0.26-8.33	3.54%
*04:05	13628 (5.65%)	324 (5.53%)	9.17E-02	2.62E-01	1.1	0.98-1.24	7.55%
*07:01	4074 (1.69%)	97 (1.66%)	3.97E-01	7.94E-01	1.1	0.89-1.36	2.15%
*08:03	17337 (7.18%)	463 (7.9%)	5.79E-01	8.28E-01	0.97	0.88-1.08	8.65%
*08:09	32 (0.01%)	1 (0.02%)	5.72E-01	8.80E-01	0.83	0.14-33.96	1.10%
*09:01	29845 (12.37%)	728 (12.43%)	5.12E-02	1.71E-01	1.08	1-1.18	15.15%
*10:01	2015 (0.84%)	57 (0.97%)	5.27E-01	8.78E-01	0.92	0.71-1.22	1.08%
*11:01	13173 (5.46%)	322 (5.5%)	2.42E-01	6.05E-01	1.07	0.96-1.21	8.59%
*12:01	6117 (2.53%)	209 (3.57%)	1.48E-04	9.88E-04	0.75	0.65-0.87	4.05%
*12:02	19159 (7.94%)	620 (10.58%)	3.95E-08	7.90E-07	0.78	0.71-0.85	9.89%
*13:01	687 (0.28%)	14 (0.24%)	4.69E-01	8.52E-01	1.28	0.76-2.36	0.38%
*13:02	4394 (1.82%)	76 (1.3%)	1.66E-04	8.28E-04	1.52	1.21-1.94	2.18%
*14:05	3360 (1.39%)	84 (1.43%)	7.43E-01	8.25E-01	1.04	0.84-1.31	2.63%
*14:54	5521 (2.29%)	197 (3.36%)	2.22E-05	2.22E-04	0.72	0.62-0.84	4.26%
*15:01	14461 (5.99%)	309 (5.27%)	2.40E-04	9.61E-04	1.24	1.1-1.4	8.34%
*15:02	3162 (1.31%)	87 (1.49%)	6.12E-01	7.65E-01	0.95	0.76-1.19	1.90%
*16:02	8573 (3.55%)	218 (3.72%)	7.54E-01	7.94E-01	1.03	0.89-1.18	5.04%

[a]*P* value for Fisher's exact test.
[b]Q value for FDR-BH adjustment.

Table S7.23. Results of HLA-DRB1 subtypes significantly associated with HBV infection in the Taiwanese population. Abbreviations: OR, odds ratio; CI, confidence interval; N, allele number.

Genotype	*HBSag-*	*HBSag+*	*P-value[a]*	*Q-value[b]*	*OR*	*95% CI*	*Allele Frequency*
	N=114,898	*N=22,632*					
*01:01	423 (0.37%)	50 (0.22%)	1.71E-05	3.11E-05	1.84	1.37-2.52	0.45%
*03:01	7781 (6.77%)	1436 (6.34%)	2.91E-09	7.27E-09	1.19	1.12-1.27	8.23%
*04:01	101 (0.09%)	18 (0.08%)	5.48E-01	6.09E-01	1.22	0.73-2.13	0.24%
*04:03	48 (0.04%)	3 (0.01%)	2.61E-02	3.26E-02	3.47	1.12-17.4	3.54%

*04:05	6694 (5.83%)	1358 (6%)	2.15E-02	2.86E-02	1.07	1.01-1.14	7.55%
*07:01	1896 (1.65%)	248 (1.1%)	1.28E-15	5.10E-15	1.67	1.46-1.92	2.15%
*08:03	8250 (7.18%)	1772 (7.83%)	7.51E-01	7.90E-01	1.01	0.96-1.07	8.65%
*08:09	16 (0.01%)	2 (0.01%)	7.57E-01	7.57E-01	1.73	0.41-15.53	1.10%
*09:01	14069 (12.24%)	3457 (15.27%)	2.85E-13	8.15E-13	0.86	0.82-0.89	15.15%
*10:01	998 (0.87%)	144 (0.64%)	1.94E-06	3.88E-06	1.51	1.26-1.81	1.08%
*11:01	6059 (5.27%)	1506 (6.65%)	8.55E-07	1.90E-06	0.86	0.81-0.91	8.59%
*12:01	2767 (2.41%)	677 (2.99%)	4.24E-03	7.06E-03	0.88	0.81-0.96	4.05%
*12:02	9131 (7.95%)	2459 (10.87%)	8.49E-24	5.66E-23	0.78	0.74-0.82	9.89%
*13:01	326 (0.28%)	13 (0.06%)	6.64E-15	2.21E-14	5.45	3.14-10.35	0.38%
*13:02	2191 (1.91%)	139 (0.61%)	5.58E-65	1.12E-63	3.48	2.93-4.17	2.18%
*14:05	1595 (1.39%)	362 (1.6%)	4.20E-01	4.94E-01	0.95	0.85-1.07	2.63%
*14:54	2587 (2.25%)	793 (3.5%)	2.96E-17	1.48E-16	0.7	0.64-0.76	4.26%
*15:01	6906 (6.01%)	1076 (4.75%)	1.50E-27	1.50E-26	1.43	1.34-1.53	8.34%
*15:02	1529 (1.33%)	284 (1.25%)	1.56E-02	2.23E-02	1.17	1.03-1.33	1.90%
*16:02	4165 (3.62%)	993 (4.39%)	5.59E-03	8.60E-03	0.9	0.84-0.97	5.04%

[a]*P* value for Fisher's exact test.
[b]Q value for FDR-BH adjustment.

Table S7.24. Results of HLA-DRB1 subtypes significantly associated with HCV infection in the Taiwanese population. Abbreviations: OR, odds ratio; CI, confidence interval; N, allele number.

Genotype	*HCVab-*	*HCVab+*	*P-value*[a]	*Q-value*[b]	*OR*	*95% CI*	*Allele Frequency*
	N=113,974	*N=10,048*					
*01:01	389 (0.34%)	36 (0.36%)	7.89E-01	9.29E-01	0.96	0.68-1.39	0.45%
*03:01	7553 (6.63%)	766 (7.62%)	2.00E-04	4.01E-03	0.86	0.79-0.93	8.23%
*04:01	99 (0.09%)	5 (0.05%)	2.80E-01	6.22E-01	1.75	0.73-5.52	0.24%
*04:03	42 (0.04%)	4 (0.04%)	7.87E-01	9.84E-01	0.93	0.34-3.57	3.54%
*04:05	6667 (5.85%)	615 (6.12%)	3.02E-01	6.05E-01	0.96	0.88-1.04	7.55%
*07:01	1739 (1.53%)	189 (1.88%)	7.87E-03	7.87E-02	0.81	0.7-0.95	2.15%
*08:03	8348 (7.32%)	693 (6.9%)	9.11E-02	3.04E-01	1.07	0.99-1.17	8.65%
*08:09	12 (0.01%)	1 (0.01%)	1.00E+00	1.00E+00	1.06	0.16-45.4	1.10%
*09:01	14568 (12.78%)	1228 (12.22%)	7.08E-02	2.83E-01	1.06	0.99-1.13	15.15%
*10:01	957 (0.84%)	91 (0.91%)	4.95E-01	9.00E-01	0.93	0.75-1.17	1.08%
*11:01	6283 (5.51%)	524 (5.21%)	1.80E-01	4.50E-01	1.07	0.97-1.17	8.59%
*12:01	2871 (2.52%)	227 (2.26%)	1.01E-01	2.88E-01	1.12	0.98-1.29	4.05%
*12:02	9673 (8.49%)	800 (7.96%)	5.15E-02	2.57E-01	1.08	1-1.17	9.89%
*13:01	288 (0.25%)	23 (0.23%)	7.55E-01	1.01E+00	1.11	0.72-1.78	0.38%
*13:02	1920 (1.68%)	206 (2.05%)	8.96E-03	5.97E-02	0.82	0.71-0.95	2.18%
*14:05	1623 (1.42%)	146 (1.45%)	8.26E-01	9.18E-01	0.98	0.83-1.18	2.63%
*14:54	2785 (2.44%)	257 (2.56%)	4.99E-01	8.32E-01	0.96	0.84-1.09	4.26%
*15:01	6614 (5.8%)	595 (5.92%)	6.85E-01	9.79E-01	0.98	0.9-1.07	8.34%
*15:02	1474 (1.29%)	138 (1.37%)	5.19E-01	7.99E-01	0.94	0.79-1.13	1.90%

*16:02	4209 (3.69%)	368 (3.66%)	8.46E-01	8.90E-01	1.01	0.91-1.13	5.04%

[a]*P* value for Fisher's exact test.
[b]Q value for FDR-BH adjustment.

Table S8.1. Results of quantiles of PRS of the target dataset. Abbreviations: OR, odds ratio; CI, confidence interval.

Quantile	*OR*	*CI.Upper*	*CI.Lower*	*N*
[0,1]	0.359553415	1.186713649	0.108938377	473
(1,5]	0.561294378	0.966649979	0.325920845	1892
(5,10]	0.722321362	1.130940544	0.461340035	2364
(10,20]	0.81282765	1.136814776	0.581175406	4728
(20,30]	0.879591326	1.225371672	0.631384679	4728
(30,40]	1.07317296	1.469586553	0.783689943	4729
(40,50]	1	1	1	4728
(50,60]	1.036937508	1.416281484	0.759198935	4728
(60,70]	1.231190325	1.663306863	0.911334913	4729
(70,80]	1.334444882	1.790418412	0.994596084	4728
(80,90]	1.345403252	1.803948629	1.003415442	4728
(90,95]	1.567832842	2.18397512	1.125516403	2364
(95,100]	1.654383368	2.295012592	1.19257922	2365

Table S8.2. Results of the performance of the PRS model in target and validate datasets. PRS-only: PRS was used in the model only. Clinical: demographics (age, sex, and BMI) and bioclinical data (albumin, HBV, and HCV) were used in model building. Combine: PRS and clinical data both were applied for building a model. Abbreviations: AUC, area under the curve; CI, confidence interval.

Dataset	*Condition*	*Accuracy (CI)*	*AUC*	*Sensitive*	*Specificity*	*Kappa*
Target	PRS-only	0.555 (0.551-0.560)	0.578	0.561	0.549	0.11
	Clinical	0.771 (0.767-0.774)	0.846	0.796	0.745	0.541
	Combine	0.775 (0.771-0.779)	0.851	0.799	0.751	0.549
Validate	PRS-only	0.546 (0.520-0.571)	0.581	0.581	0.543	0.029
	Clinical	0.594 (0.568-0.618)	0.842	0.919	0.574	0.116
	Combine	0.611 (0.586-0.636)	0.843	0.895	0.594	0.119

Table S8.3. Results of the performance by using PRS, clinical data, and combined both to predict the risk of the high PRS group for HCC development. Abbreviations: AUC, area under curve; CI, confidence interval.

Top of PRS	*Model*	*AUC (95% CI)*	*N*
>=95%	PRS-only	0.686 (0.501-0.870)	90
	Clinical	0.857 (0.724-0.991)	
	Combine	0.811 (0.672-0.949)	
>=90%	PRS-only	0.565 (0.390-0.739)	165
	Clinical	0.855 (0.750-0.959)	
	Combine	0.831 (0.726-0.973)	
>=85%	PRS-only	0.496 (0.337-0.655)	240
	Clinical	0.854 (0.758-0.950)	
	Combine	0.824 (0.714-0.934)	
>=80%	PRS-only	0.468 (0.320-0.615)	315
	Clinical	0.827 (0.732-0.921)	
	Combine	0.802 (0.699-0.905)	

Table S9. PheWAS Results by HCC PRS in Taiwanese.

**description**	**group**	**q-val**
Viral hepatitis B	infectious diseases	8.92E-03
Viral hepatitis	infectious diseases	1.25E-03
Cancer of liver and intrahepatic bile duct	neoplasms	1.09E-02
Althete's foot	infectious diseases	7.87E-04
Acute, but ill-defined cerebrovascular disease	circulatory system	2.73E-03

Table S10. Results of PRS distribution among different family cancer groups. Abbreviation: mPRS, mean of PRS.

Type	*5 Group*		*Type*	*3 Group*	
	N	*mPRS (SD)*		*N*	*mPRS (SD)*
Liver	798	-0.150862 (0.988)	Liver	1358	-0.110848 (0.988)
Liver+	560	-0.053828 (0.987)			
Other	3980	-0.235087 (1.014)	Other	5560	-0.232855 (1.009)
Other+	1580	-0.227234 (0.995)			
Healthy	11665	-0.181311 (1.004)	Healthy	11665	-0.181311 (1.004)
